# A joint model for the estimation of species distributions and environmental characteristics from point-referenced data

**DOI:** 10.1371/journal.pone.0304942

**Published:** 2024-06-21

**Authors:** Markus Viljanen, Lisa Tostrams, Niels Schoffelen, Jan van de Kassteele, Leon Marshall, Merijn Moens, Wouter Beukema, Wieger Wamelink

**Affiliations:** 1 Department of Statistics, Data Science and Modelling, National Institute for Public Health and the Environment, Bilthoven, Utrecht, The Netherlands; 2 Centre for Environmental Quality, National Institute for Public Health and the Environment, Bilthoven, Utrecht, The Netherlands; 3 Naturalis Biodiversity Center, Leiden, South-Holland, The Netherlands; 4 Reptile, Amphibian & Fish Conservation Netherlands (RAVON), Nijmegen, Gelderland, the Netherlands; 5 Wageningen Environmental Research, Wageningen University & Research, Wageningen, Gelderland, The Netherlands; 6 Agroecology Lab, Interfaculty School of Bioengineering, Université libre de Bruxelles (ULB), Brussels, Région de Bruxelles-Capitale, Belgium; University of Deusto: Universidad de Deusto, SPAIN

## Abstract

**Background:**

Predicting and explaining species occurrence using environmental characteristics is essential for nature conservation and management. Species distribution models consider species occurrence as the dependent variable and environmental conditions as the independent variables. Suitable conditions are estimated based on a sample of species observations, where one assumes that the underlying environmental conditions are known. This is not always the case, as environmental variables at broad spatial scales are regularly extrapolated from point-referenced data. However, treating the predicted environmental conditions as accurate surveys of independent variables at a specific point does not take into account their uncertainty.

**Methods:**

We present a joint hierarchical Bayesian model where models for the environmental variables, rather than a set of predicted values, are input to the species distribution model. All models are fitted together based only on point-referenced observations, which results in a correct propagation of uncertainty. We use 50 plant species representative of the Dutch flora in natural areas with 8 soil condition predictors taken during field visits in the Netherlands as a case study. We compare the proposed model to the standard approach by studying the difference in associations, predicted maps, and cross-validated accuracy.

**Findings:**

We find that there are differences between the two approaches in the estimated association between soil conditions and species occurrence (correlation 0.64-0.84), but the predicted maps are quite similar (correlation 0.82-1.00). The differences are more pronounced in the rarer species. The cross-validated accuracy is substantially better for 5 species out of the 50, and the species can also help to predict the soil characteristics. The estimated associations tend to have a smaller magnitude with more certainty.

**Conclusion:**

These findings suggests that the standard model is often sufficient for prediction, but effort should be taken to develop models which take the uncertainty in the independent variables into account for interpretation.

## 1 Introduction

Biodiversity worldwide is under threat by a multitude of stressors, including anthropogenic pressures such as pollution and climate change [1, 2]. Successfully and effectively mitigating threats to a given species requires knowledge about its ecology, but such information is not always available. One solution is to use ecological models to explain and predict species presence.

A powerful tool to this aim are Species Distribution Models (SDMs), which are statistical models that attempt to predict and explain species occurrence using environmental characteristics [3, 4]. The response variable is species occurrence and the explanatory variables are most often environmental characteristics that include various descriptors of the abiotic environment. Researchers have developed increasingly complex SDM techniques based on statistical models and machine learning [5, 6]. SDMs are fitted to spatial data, where spatial auto-correlation is a characteristic that should be taken into account for statistical inference [7, 8] and prediction [9–11]. For more complete description of SDMs and related statistical issues we refer the reader to reviews [12, 13].

Data sets that describe environmental conditions are becoming increasingly available [14]. More data offers promise in more fully capturing the habitat characteristics of species, which can result in more accurate maps and detection of novel predictors of species occurrence [15–17]. These predictors are readily used in SDMs or other ecological models. However, environmental data is often predicted from other models, measured with error, or interpolated from measurement points. There is inherent uncertainty in GIS layers [18–20], local climate interpolated from weather stations [21, 22], thematic resolution and change of land use [23], and the coordinates of species occurrence in historical data [24]. Recent studies suggest that poor model performance can be attributed to high levels of uncertainty in the environmental data [25]. Spatial misalignment, where the measurements of environmental factors are not properly aligned to the species observation data, is one critical source of uncertainty in studying the effect of environmental factors on species distribution [26].

Predicting an accurate map of habitat suitability requires accurate environmental conditions at every possible point of the study region. A simple solution to unknown values is a two-stage approach. In the first stage, one predicts the environmental factor at every spatial location. Typical solutions are using a geostatistical model such as kriging, a machine learning model such as random forest, or down scaling each observation into a full coverage grid. At the second stage, these predicted environmental factors are treated as ground truth in the species distribution model. However, this approach does not consider uncertainty in the covariate values, which can lead to erroneous statistical inferences [27]. Few studies have attempted to assess the effect of uncertainty in the environmental variables on an SDM model [26–32]. There is still a need for a proper method for realistic SDMs that combines errors from the different environmental variables into a single model [33, 34].

One solution is to implement so-called errors-in-variables models [35]. Both classical measurement error and Berkson errors can be considered in a species distribution model depending on the beliefs about the underlying phenomena [31]. The models can be estimated by integrating over the latent variables without error in either frequentist (EM-algorithm) or Bayesian (MCMC or INLA) statistical inference. It is difficult to develop a fully general model because a complex species distribution model has to be integrated with an extrapolation method for the predictors to correctly consider the uncertainty and its propagation. Here, we propose a hierarchical Bayesian model as a particularly attractive solution, because both the observed data and model parameters can be random variables in Bayesian inference [36]. Approximations used by INLA make it possible to fit complex hierarchical and spatial models to large data sets [37]. Standard SDMs have been implemented before in the Bayesian framework using the INLA package, see for example [38–42]. As a novelty, we extend this approach to consider the environmental factors from a set of models as input to the SDM, with all models estimated jointly from data.

In this paper, we implement the joint model and the standard two-stage model in a Bayesian framework with a specification that is otherwise identical. Our aim is to investigate the impact of taking the uncertainty in the predictor variables into account. We compare the estimated associations, predicted maps, and accuracy in cross-validated data. Since there is uncertainty inherent in any model, including maps and associations predicted from an SDM, it is best if the uncertainty is explicitly quantified so that well-justified decisions can be made [33, 43–45].

## 2 Data

As a use case, we consider the Netherlands where The National Flora Monitoring Network—Environment and Nature Quality (LMF-M&N) [46] collects a data set of species observations and the Wageningen University & Research [47] has measured abiotic factors. Based on these data sets, we build SDMs to illustrate how uncertainty in the abiotic factors can be incorporated into the species distribution model.

The LMF-M&N [46] aims to collect measurements to inform policy makers in the Netherlands. One interest of the national government is to conserve the biodiversity in natural areas. Less common native plant species are threatened in particular and are an important consideration for making policy for nature management [48]. The data set is an inventory of vegetation in permanent test areas, recorded using the Braun-Blanquet method [49]. We considered the data recorded across 12799 plots up until 2019. All species of vascular plants (Tracheophytes) present in these plots are noted during field visits, and we extract the presence/absence of every species at every plot. In total 1442 plant species were observed. The ecologists performing the observations were given a list of possible plant species that could be found in this region, along with a list of species that had been observed at this plot in the past. In general, the recordings at any plot are repeated once every four years. The size of the plots differed per vegetation type that it falls in, generally ranging from between 2x2m and 5x5m for grasslands, and between 25x25m and 50x50m for forests. The monitoring network is a stratified sample, where natural areas of interest are over-represented in sampling. The Netherlands was divided into smaller spatial units [46] based on physical geographical regions (FGR) sharing similar environmental characteristics [50], which were further refined into smaller areas with different vulnerability to acidification and eutrophication [51].

Abiotic environmental factors are available for a subset of plots in a database from Wageningen University & Research [47]. Field measurements are used to record soil conditions such as pH, groundwater table, potassium and nitrogen at 1208 plots. Not every measurement was available at every location in the original dataset, but the 6 soil variables extracted from this data set were recorded at most plots. The abiotic data set is collected independently from the LMF-M&N but can be linked to it using the spatial location of each plot. However, this results in spatial misalignment where some species plots fully overlap with the abiotic field measurements, others are close by to some measurement, and others are far away. This results in varying degrees of uncertainty about abiotic factors at each species plot.

**Fig 1:**
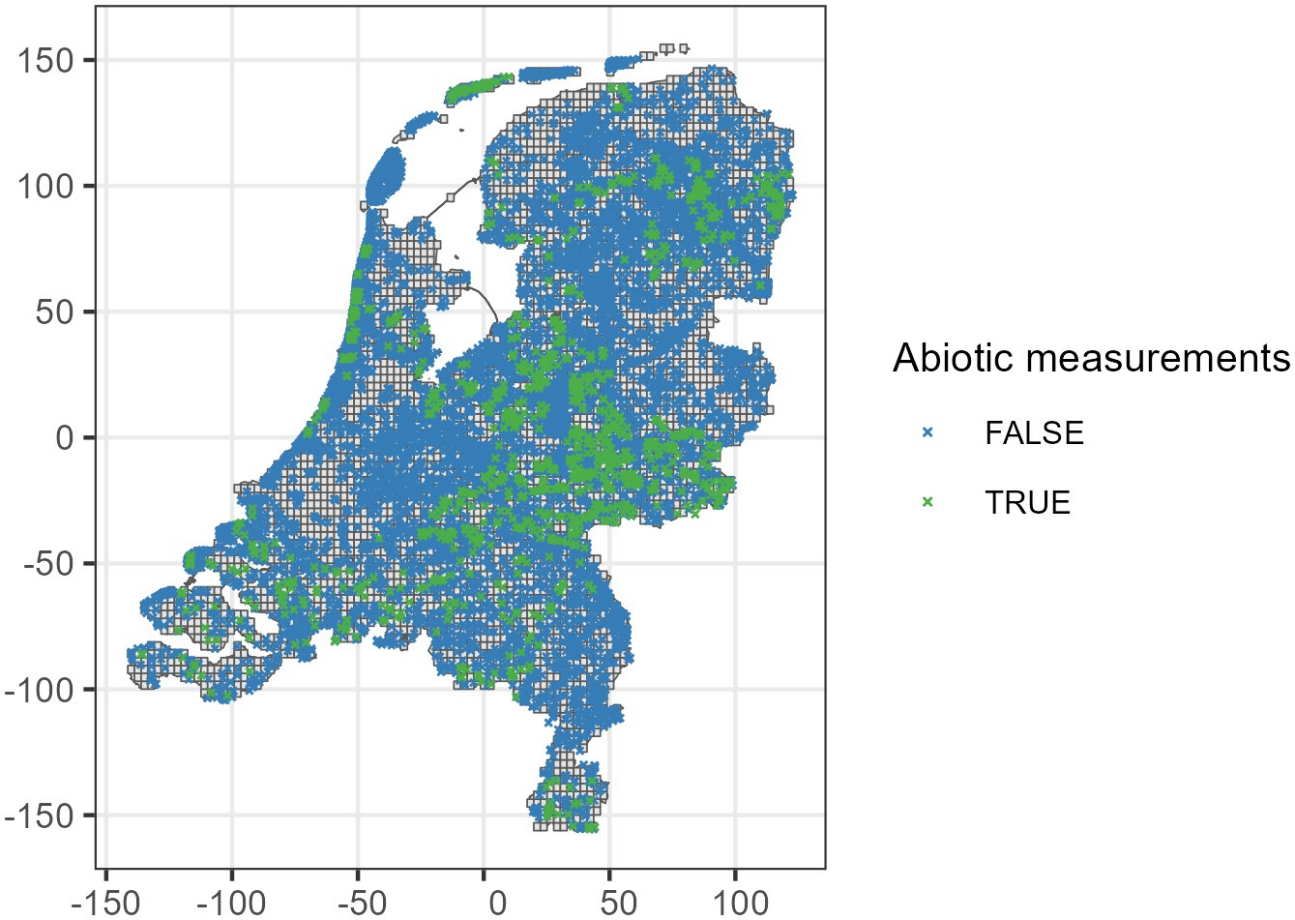
Location of field visits for vegetation survey and abiotic measurements in the Netherlands. The blue dots show the location of species observations in LMF-M&N dataset, while the green dots represent the abiotic measurements from Wageningen University & Research. A grid of 2.88km x 2.88km cells has been created and imposed on the Netherlands to define the grid cell centre as the location of predictions, but its resolution can be adjusted arbitrarily. The axis denote distance to the centre of the Dutch coordinate system (Rijksdriehoek) in km.

The spatial misalignment is illustrated in Fig 1 and Table 1. The Netherlands spans an extent of 312km x 264km and we created a grid of 2.88km x 2.88km cells to define the location of predictions in a full-coverage map, the resolution of which could be arbitrarily increased or decreased. Note in Fig 1 that, even if the abiotic measurements (green) and species occurrence field visits (blue) would have been perfectly aligned, predicting the entire Netherlands would also require the measurement of abiotic variables in all grid points. In principle, similar partial overlap and uncertainty is present when considering the year of measurement. For simplicity, we take the latest field visit and focus on spatial uncertainty.

**Table 1:** Total number of distinct species, plots, and years in data sets. The (plot, year)-pairs define a distinct field visit or abiotic measurement in space and time.

Data	Species	Plot	Year	(Plot,Year)	Variables
LMF-M&N	1442	12799	21	52302	1
Abiotic Variables		1208	5	1208	6

We have chosen eight independent variables that describe habitat preferences in the SDM: day number of field visit, pH, amount of Organic matter (ORG), Carbon/Nitrogen-Ratio (C/N), Nitrogen (N), Phosphorus (P), Potassium (K), and land use [52]. Land use classification is available for the entire Netherlands from open spatial data. The day number, pH and land use are considered known, the five soil conditions ORG, C/N, N, P, K are considered uncertain over the entire Netherlands. For modelling these variables, we have log transformed them and converted them to z-scores, i.e. mean centered and divided by the standard deviation, to ensure that the vague prior has an equal impact on all variables. In addition, location information is provided as x&y-coordinates based on the ‘Rijksdriehoekscoördinaten’, the Dutch coordinate system. Every point has x-coordinate between 0 and 280 km and y-coordinate between 300 and 625 km, where the central point of the projection is in Amersfoort x = 155 km, y = 463 km. In the model and produced maps, we expressed both the x-coordinate and the y-coordinate as a deviation from the central point in km.

The day number (1–365) of field visit is recorded at every field visit, so it is available at each location. Most observations were done between March and November. We use an interpolated pH map in S1 Fig, which was created from pH measurements with kriging using the following covariates: land use, soil type [53], and common heather (*Calluna vulgaris*) as an indicator plant. Research indicates that similar maps of pH in the natural areas of Netherlands can be considered accurate [54]. We re-coded landuse into categories: Forest: -4, Dry natural terrain: -3, Wet natural terrain: -2, Water: -1, Recreation: 0, Railway: 1, Main road: 2, Build: 3, Agriculture and other agricultural: 4. Land use is illustrated in S2 Fig and the number of plots was stratified towards natural areas as illustrated in S3 Fig.

In total 50 species were selected by hand, see S1 Table. Criteria for the selection, besides a minimum number of positive findings of 20 in the database, were that the selection should be representative for the Dutch flora in natural areas. This means that we selected rare species according to the national Red List, threatened species, common species and species increasing in occurrence. Also some tree species, shrubs and dwarf shrubs were included in the selection as well as the most common species in the dataset *Holcus lanatus*. Species selection reflects a variety in preferences for abiotic circumstances, from nutrient poor to nutrient rich and from dry to wet.

## 3 Methods

We compare two models that both implement spatial interpolation combined with a species distribution model in a Bayesian framework and apply these to all of the 50 selected species:

1. Two-stage model: Model the abiotic variables, then fit a species distribution model to the predicted mean of abiotic variables.
2. Joint model: Model the abiotic variables and species distribution simultaneously, by using unknown latent abiotic variables.

These two approaches contain identical model components. One standard kriging model is used for every abiotic variable. A standard generalized additive model with a spatial component is used for the species distribution model based on these abiotic variables. The only difference is that the joint model does not assume the abiotic values are known for certain. The two stage model uses the predicted mean abiotic values at each species observation, whereas the joint model considers the entire possible distribution of the abiotic variables. This is accomplished by chaining the two models together in a hierarchical Bayesian model by feeding the output of abiotic variable models as input to the species distribution model, which are then random variables instead of constants. The proposed species distribution model is not based on the potentially false assumption of accurately measured abiotic variables. In the next 3 sections we first define the kriging model, then the SDM, and finally how they are combined in these two approaches.

### 3.1 Interpolation of abiotic variables

Kriging refers to a widely used method for interpolation or smoothing spatial data [55]. For a model-based approach, we assume data to be generated by a stochastic process, which is a real-valued random variable X*(***a***) indexed by a point ***a*** in domain $D\subseteq R^{2}$. Given abiotic measurement locations $D={\left\{a_{i}\right\}}_{i=1}^{M}$, the abiotic measurements consist of realizations of the process at these locations:

$$\begin{array}{c} x^{*}=\left(x^{*}\left(a_{1}\right),\ldots ,x^{*}\left(a_{M}\right)\right) \end{array}$$ (1)

The stochastic process is a Gaussian Random Field (GRF) if for a set of locations the outcome vector is assumed to have a multivariate Normal distribution x* ∼ MVN(***μ***, Σ) where ***μ***_*i*_ = E[X*(***a***_*i*_)] and $\Sigma_{i,j}=C\left(X^{*}\left(a_{i}\right),X^{*}\left(a_{j}\right)\right)$.

A model specifies a probability distribution for the vector of observed outcomes x* from the stochastic process X*(***a***), which we consider a ‘noisy’ realization of the latent Gaussian Field (GF) *X*(***a***). We define the observed stochastic process as X*(***a***) = *X*(***a***) + *ϵ* where *ϵ* ∼ N(0, *δ*^2^) is the measurement error. The underlying latent field for an abiotic variable is specified by a mean value E[*X*(***a***_*i*_)] = *α* and the Matérn covariance function:

$$\begin{array}{c} C\left(X\left(a_{i}\right),X\left(a_{j}\right)\right)=\frac{\sigma^{2}}{2^{\nu -1}\Gamma \left(\nu \right)}\left(\kappa \right\|a_{i}-a_{j}{\left\|\right)}^{\nu }K_{\nu }\left(\kappa \right\|a_{i}-a_{j}\left\|\right) \end{array}$$ (2)

where we used INLA’s default smoothness parameter *ν* = 1 and *K*_*ν*_ is the modified Bessel function of the second kind of order *ν*. The parameter *σ*^2^ is the variance and *κ* is the scaling parameter of the spatial effect. The range is the distance where the spatial correlation is close to zero, related to the scaling parameter and empirically defined as $r=\frac{\sqrt{8}}{\kappa }$ for the Matérn covariance function.

The covariance function C now depends on two parameters: the variance (partial sill in kriging) and the range of the spatial effect. For large data sets, it can be computationally infeasible to estimate a dense spatial covariance matrix Σ. This is known as the ‘big n’ problem [56]. A computationally effective alternative is given by the stochastic partial differential equation (SPDE) approach [57], which involves a sparse precision matrix. The internal SPDE approach is parameterised by *τ* and *κ* and *σ*^2^ then follows from $\sigma^{2}=\frac{1}{8\pi \kappa^{2}\tau^{2}}$. The default prior is log(*τ*) ∼ N(0, 10) and log(*κ*) ∼ N(0, 10).

The intercept defined as a fixed effect has a default prior α ∼ N(0, 1000). For the measurement error the default prior is defined in terms of precision, i.e. inverse of the variance $\frac{1}{\delta^{2}}∼\text{Gamma}\left(1,10^{-5}\right)$.

### 3.2 Species Distribution Model (SDM)

We specify the SDM as a Generalized Additive Mixed Model (GAMM). We model species occurrence at a given location by a binary random variable *Y*(***s***), indexed by a point ***s*** in domain $D^{\prime }\subseteq R^{2}$. Given species observation locations $D^{\prime }={\left\{s_{i}\right\}}_{i=1}^{N}$, the outcomes are presence/absence at these locations:

$$\begin{array}{c} y=(y\left(s_{1}\right),\ldots ,y\left(s_{N}\right)) \end{array}$$ (3)

Let *y*(***s***_*i*_) denote the observed values of the binary random variable *Y*(***s***_*i*_) ∼ Bernoulli(*μ*_*i*_) whether the species is observed at location *i*, where $\mu_{i}=g\left(\eta_{i}\right)=\frac{1}{1+e^{-\eta_{i}}}$ is the probability of occurrence at plot *i* given by the logistic link function. A structured additive predictor *η*_*i*_ accounts for the covariates in an additive way:

$$\begin{array}{c} \eta_{i}=\beta_{0}+\sum_{j=1}^{K}\;\beta_{j}x_{j}\left(s_{i}\right)+\sum_{j=1}^{L}\;f_{j}\left(z_{j}\left(s_{i}\right)\right) \end{array}$$ (4)

where *β*_0_ is the intercept; $\beta =(\beta_{1},\ldots ,\beta_{K})$ are the coefficients with linear relationship to covariates ***x*** = (*x*_1_, …, *x*_*K*_) and ***f*** = (*f*_1_, …, *f*_*L*_) are functions of the covariates ***z*** = (*z*_1_, …, *z*_*L*_). These can be non-linear effects of covariates, time trends, random intercepts and slopes, temporal or spatial random effects. Non-linear effects can be modelled using a spline *f*_*j*_(***u***) based on a random walk model of order 2 over discrete domain ***u*** = (*u*_1_, …, *u*_*d*_) of the covariate values, where the second order increments are distributed as Δ^2^*u*_*i*_ = *u*_*i*_ − 2*u*_*i*+1_ + *u*_*i*+2_ ∼ N(0, *ζ*^2^). We use splines for day number, pH, and land use, while ORG, C/N, N, P, K are entered as fixed effects. The fixed effects, including the intercept, have default priors *β*_*j*_ ∼ N(0, 1000). The default prior on random walk spline is again defined in terms of precision, i.e. inverse of the variance $\frac{1}{\zeta^{2}}∼\text{Gamma}\left(1,10^{-5}\right)$.

### 3.3 Two-stage and joint models

The two-stage and joint models include the same two modelling tasks; interpolation of abiotic variables and SDM. There are two problems that cause uncertainty about the true covariate values in the SDM:

1. We observe a realization $x_{j}^{*}\left(s\right)=x_{j}\left(s\right)+\epsilon$ with an unknown amount of measurement error *ϵ* > 0.
2. The set of abiotic measurements D does not contain every location $R^{2}$, so $x_{j}^{*}\left(s\right)$ is unobserved for s∉D.

We have noisy observations $x_{j}^{*}\left(s\right)$ at every abiotic measurement location s∈D. However, the SDM defined in the previous section assumes the underlying realization of the random field *x*_*j*_(***s***) of every covariate *j* is known at every field visit location $s\in D^{\prime }$ for fitting the model and entire Netherlands $s\in R^{2}$ for predicting the map. Because the true abiotic values are not known, we must define how an SDM is fitted to the noisy point-referenced data.

First, consider *K* spatial (kriging) models for each covariate:

$$\begin{array}{c} \begin{array}{cc} X_{1}^{*}\left(s\right) & =X_{1}\left(s\right)+\epsilon_{1}\\ X_{1}\left(s\right) & ∼\text{MVN}(\alpha_{1},\Sigma \left(\sigma_{1}^{2},r_{1}\right))\\ \epsilon_{1} & ∼N(0,\delta_{1}^{2})\\ {\hat{x}}_{1}\left(s\right) & =E[X_{1}\left(s\right)|{\left\{x_{1}^{*}\left(a_{i}\right)\right\}}_{i=1}^{M}]\\ & \cdots \\ X_{K}^{*}\left(s\right) & =X_{K}\left(s\right)+\epsilon_{K}\\ X_{K}\left(s\right) & ∼\text{MVN}(\alpha_{K},\Sigma \left(\sigma_{K}^{2},r_{K}\right))\\ \epsilon_{K} & ∼N(0,\delta_{K}^{2})\\ {\hat{x}}_{K}\left(s\right) & =E[X_{K}\left(s\right)|{\left\{x_{K}^{*}\left(a_{i}\right)\right\}}_{i=1}^{M}] \end{array} \end{array}$$ (5)

where the random variable $X_{j}^{*}\left(s\right)$ is a noisy realization of covariate *X*_*j*_(***s***) with additional variance $\delta_{j}^{2}$ in the outcome to express any possible measurement error. The underlying random field for each covariate *j* is specified as a realization of the Gaussian process depending on intercept *α*_*j*_ and spatial correlation parameters $\sigma_{j}^{2},r_{j}$. We have also defined predicted abiotic values ${\hat{x}}_{j}\left(s\right)$ as the the expected value of the posterior predictive distribution. These predictions are made at every field visit location $D^{\prime }={\left\{s_{i}\right\}}_{i=1}^{N}$ given observations at abiotic measurement locations $D={\left\{a_{i}\right\}}_{i=1}^{M}$.

The two-stage model uses the predicted abiotic values as known constants:

$$\begin{array}{c} \begin{array}{cc} Y(s_{i}) & ∼\text{Bernoulli}(\mu_{i})\\ \mu_{i} & =\frac{1}{1+e^{-\eta_{i}}}\\ \eta_{i} & =\beta_{0}+\beta_{1}{\hat{x}}_{1}\left(s_{i}\right)+\ldots +\beta_{K}{\hat{x}}_{K}\left(s_{i}\right)+\\ & f_{1}\left(z_{1}\left(s_{i}\right)\right)+\ldots +f_{L}\left(z_{L}\left(s_{i}\right)\right)+W\left(s_{i}\right)\\ W(s) & \;∼\text{MVN}(0,\Sigma \left(\sigma_{0}^{2},r_{0}\right)) \end{array} \end{array}$$ (6)

The joint model considers the abiotic values *x*_*j*_(***s***) in every location as unknown latent variables *X*_*j*_(***s***):

$$\begin{array}{c} \begin{array}{cc} Y(s_{i}) & ∼\text{Bernoulli}(\mu_{i})\\ \mu_{i} & =\frac{1}{1+e^{-\eta_{i}}}\\ \eta_{i} & =\beta_{0}+\beta_{1}X_{1}\left(s_{i}\right)+\ldots +\beta_{K}X_{K}\left(s_{i}\right)+\\ & f_{1}\left(z_{1}\left(s_{i}\right)\right)+\ldots +f_{L}\left(z_{L}\left(s_{i}\right)\right)+W\left(s_{i}\right)\\ W(s) & \;∼\text{MVN}(0,\Sigma \left(\sigma_{0}^{2},r_{0}\right)) \end{array} \end{array}$$ (7)

**Fig 2:**
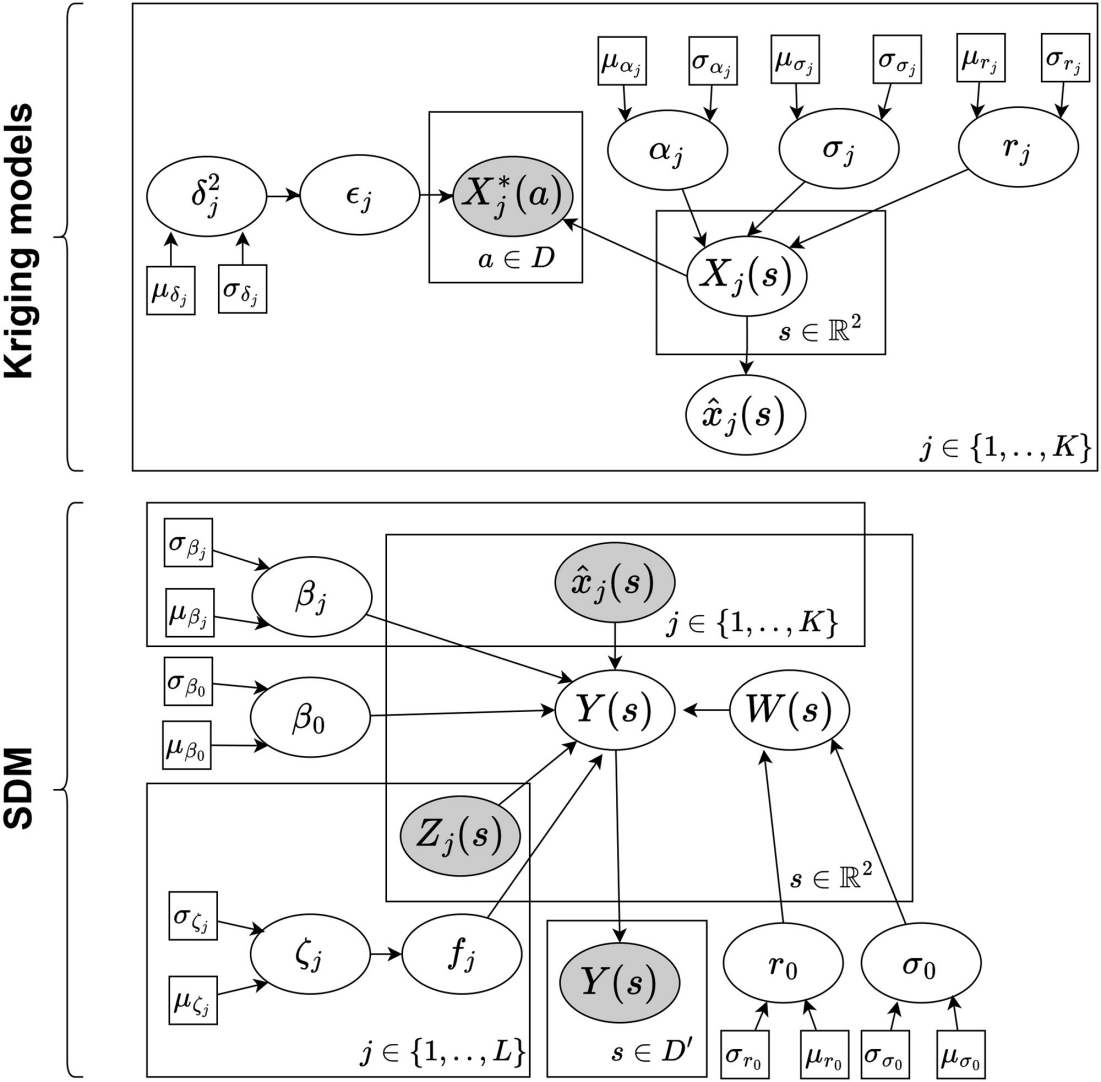
Directed Acyclic Graph representation in plate notation of the two-stage Bayesian model which first uses standard kriging models to spatially interpolate abiotic variables, and then fits a Species Distribution Model (SDM) to the predicted abiotic variables. A rectangle is used to group variables that repeat together with the repeating subscript or argument defined at the bottom right. The white nodes are random variables, the gray nodes are observations of random variables, the square nodes are fixed values corresponding to hyperparameters. In the kriging models, $X_{j}^{*}\left(a\right)$ are the observed values for the abiotics at locations *a* ∈ *D*, *X*_*j*_(*s*) are the random variables depicting the random field at all locations $s\in R^{2}$, and ${\hat{x}}_{j}\left(s\right)$ are the posterior means, which can be seen as predicted abiotic values, for all *K* abiotic variables. In the SDM, ${\hat{x}}_{j}\left(s\right)$ are treated as observations of true abiotic values, *Z*_*j*_(*s*) are the non-linear covariates, *W*(*s*) is the spatial effect, and *Y*(*s*) is the binary random variable indicating whether a species occurs at all locations $s\in R^{2}$ with observations at locations *s* ∈ *D*′. This two-stage model disregards the uncertainty about the true abiotic values. For the formulae refer to Eqs 5 and 6.

**Fig 3:**
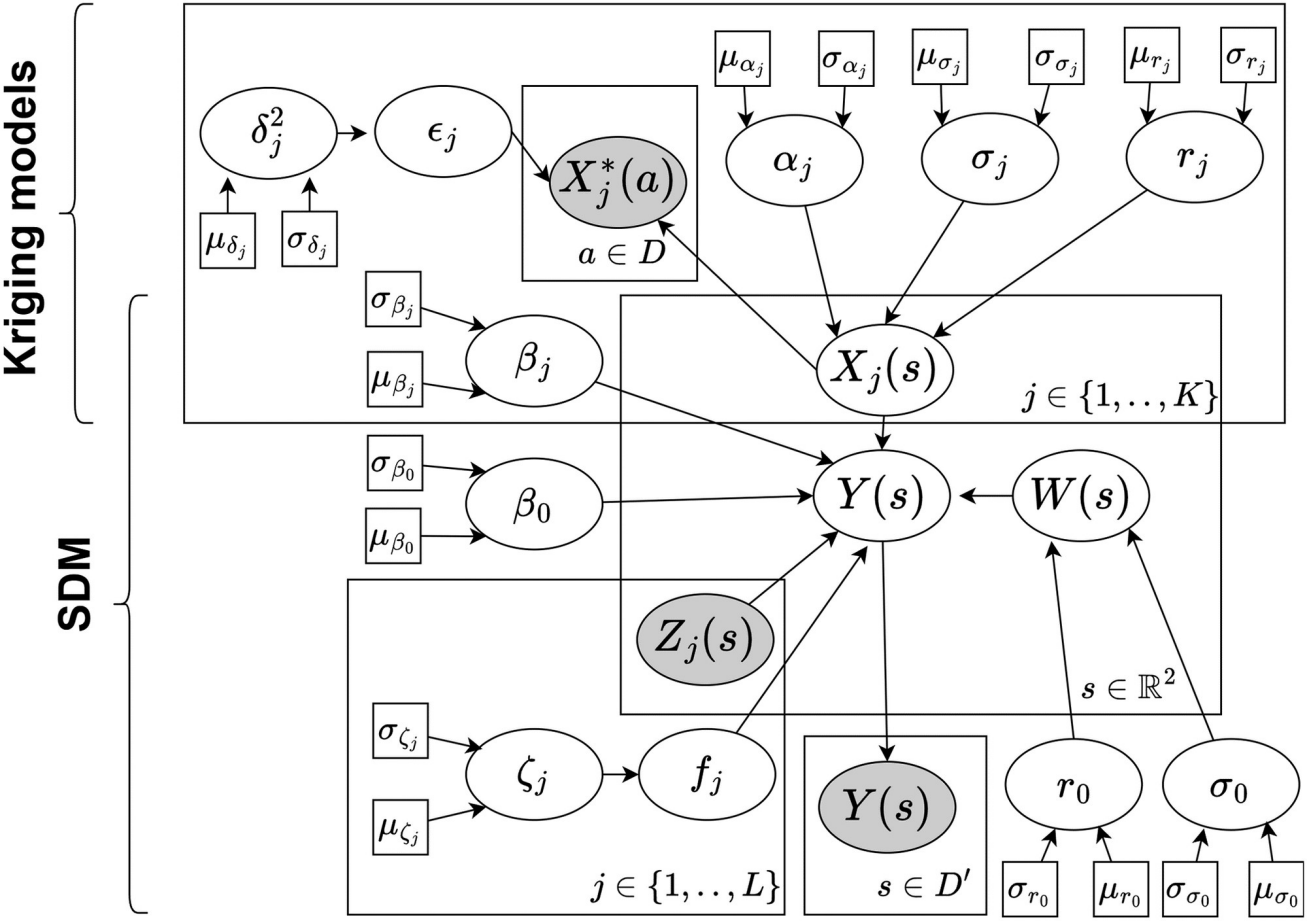
Directed Acyclic Graph representation in plate notation of the two-stage Bayesian model which first uses standard kriging models to spatially interpolate abiotic variables, and then fits a Species Distribution Model (SDM) to the predicted abiotic variables. A rectangle is used to group variables that repeat together with the repeating subscript or argument defined at the bottom right. The white nodes are random variables, the gray nodes are observations of random variables, the square nodes are fixed values corresponding to hyperparameters. In the kriging models, $X_{j}^{*}\left(a\right)$ are the observed values for the abiotics at locations *a* ∈ *D*, *X*_*j*_(*s*) are the random variables depicting the random field at all locations $s\in R^{2}$, for all *K* abiotic variables. In the SDM, the latent random variable *X*_*j*_(*s*) describes the true abiotic values, *Z*_*j*_(*s*) are the non-linear covariates, *W*(*s*) is the spatial effect, and *Y*(*s*) is the binary random variable indicating whether a species occurs at all locations $s\in R^{2}$ with observations at locations *s* ∈ *D*^′^. The abiotic random variable is linked directly to the SDM, which means the uncertainty is taken into account by considering the entire distribution of the likely values. For the formulae refer to Eqs 5 and 7.

In both models, *μ*_*i*_ is the probability of occurrence at field visit *i* = 1, …, *N* given by the logistic link function *g*(*η*_*i*_). At each field visit *i*, we have *K* random variable covariates ${\left\{X_{j}\left(s_{i}\right)\right\}}_{j=1}^{K}$ with linear effects and *L* fixed covariates ${\left\{z_{j}\left(s\right)\right\}}_{j=1}^{L}$ with non-linear effects *f*_*j*_ modelled by a spline based on a random walk model of order 2. The models also include a spatial effect *W*(***s***) to model any spatial patterns in species occurrence unaccounted by the covariates. We use a Directed Acyclic Graph (DAG) to illustrate the two-stage model in Fig 2 and the joint model in Fig 3.

The two-stage model fits the *K* independent kriging models first, and uses the resulting predictions as input to a species distribution model fitted afterwards. Note that the joint model combines *K* + 1 separate models: *K* spatial models for each covariate and 1 species distribution model based on these covariates. These models are fitted simultaneously to observed data in each location, and it is not a requirement that every covariate or the species is observed at every location. The joint model correctly propagates all uncertainty related to the covariate prediction but can be computationally expensive especially for *K* > 1.

### 3.4 Validation

**Fig 4:**
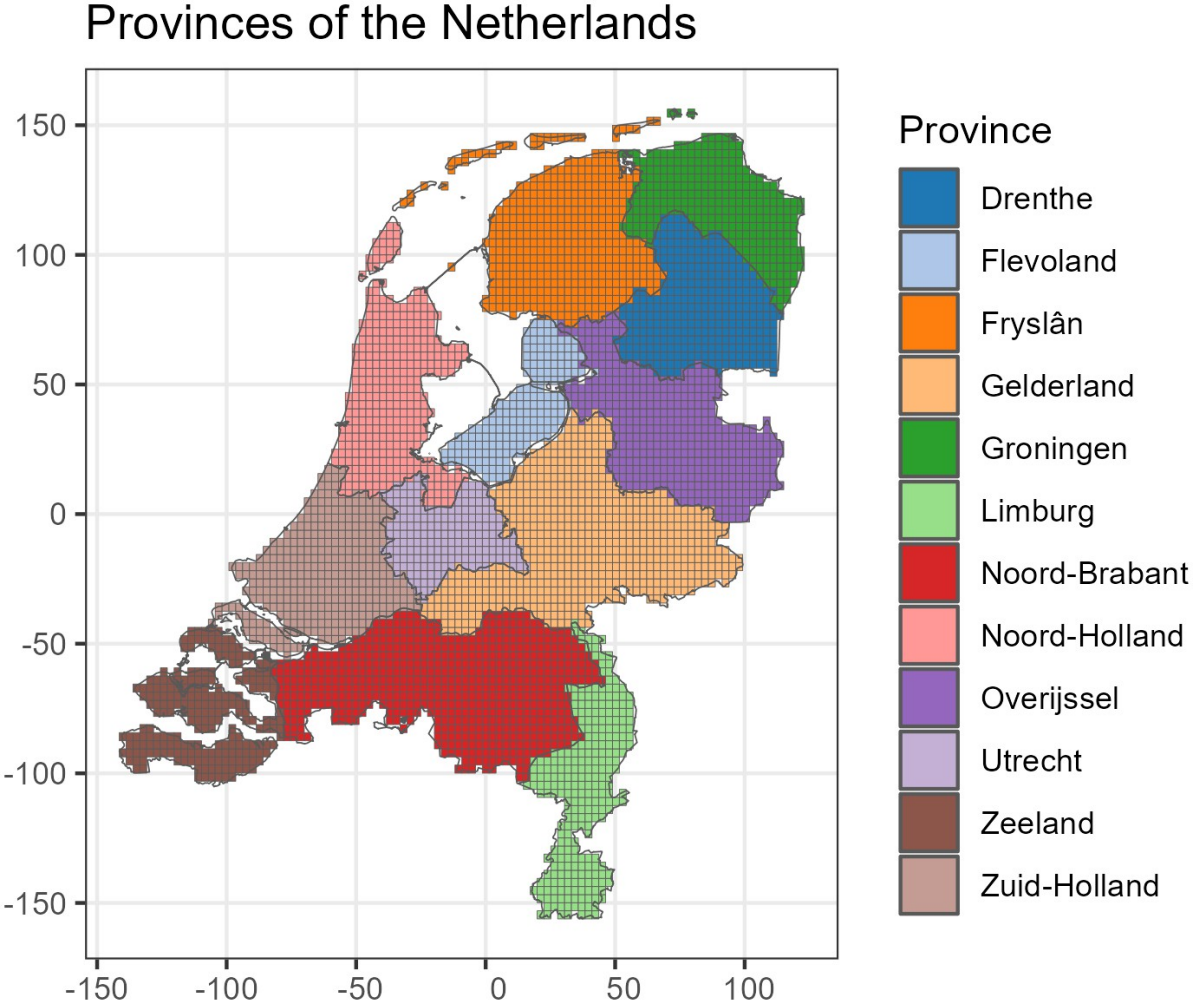
The Netherlands subdivided into provinces. These divisions were used for leave-province-out cross validation, where one province is left out for validation and the model is trained on other provinces, the process is then repeated for each province.

**Fig 5:**
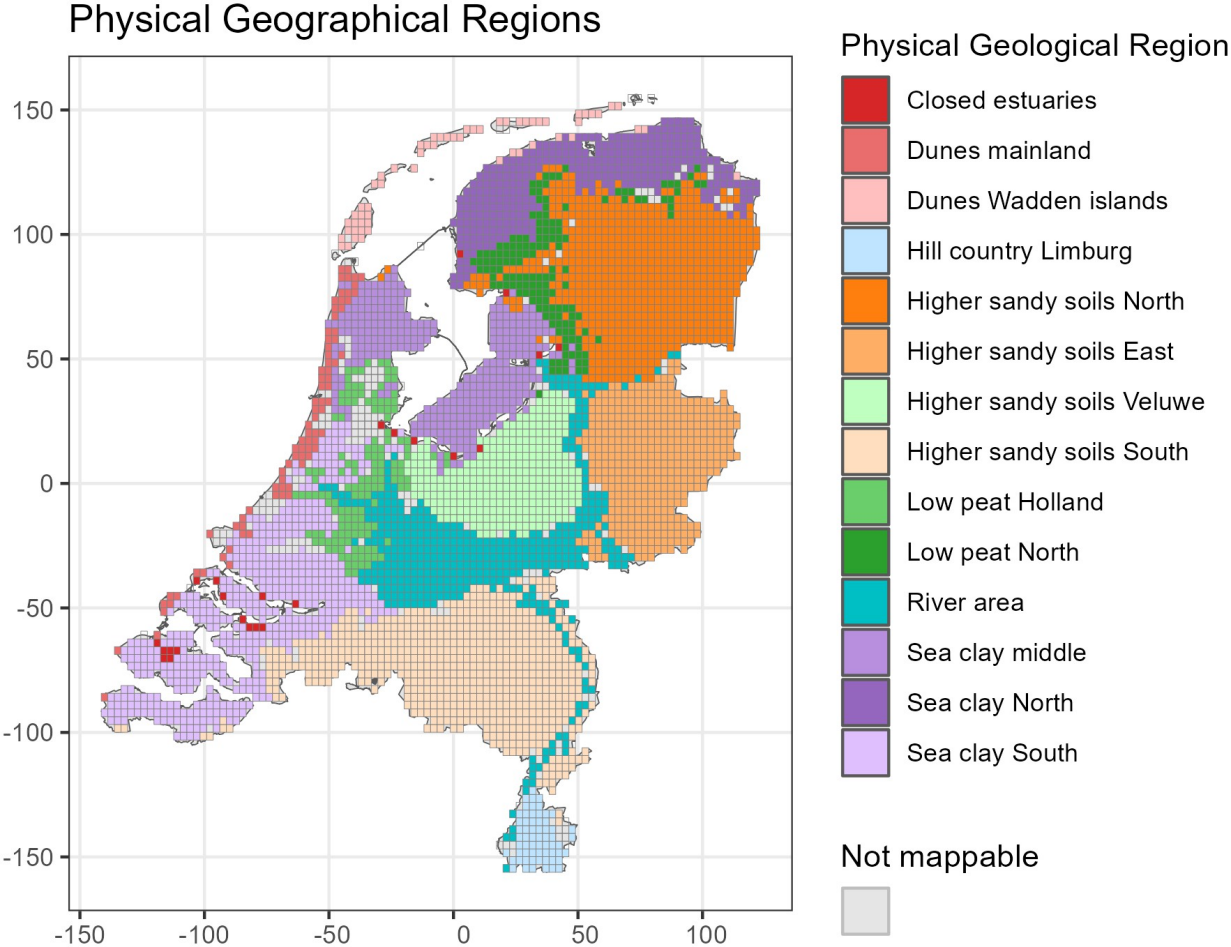
The Netherlands subdivided into physical geographical regions (FGRs). These divisions were used for leave-region-out cross validation, where one region is left out for validation and the model is trained on other regions, the process is then repeated for each region.

In order to evaluate model generalization ability, we mimic a scenario in which practitioners must use a model that has been trained in one area to estimate species occurrence in another location. We use spatial cross validation for the validation sets due to spatial correlation, since otherwise it would be relatively easy to predict species occurrence from observed nearby species occurrence. We formed the validation sets using both the set of provinces of the Netherlands as well as the Dutch Physical Geographical Regions (FGR) [50], see Figs 4 and 5. Provinces are administrative regions so we have some overlap in their environmental characteristics, which may help the model to estimate the habitat suitability of every species. The FGRs are regions defined by shared environmental characteristics, so we expect more errors since there are fewer similar areas included in the train data set.

The models are trained by leaving all field visits in one of the regions out from training data and training on all other field visits. The predicted posterior distribution of the species occurrence probability *μ*_*i*_ ∈ [0, 1] is then compared to the true species occurrence *y*_*i*_ ∈ {0, 1} in validation data. This gives cross-validated predictions for every species and field visit. We report the model accuracy as the Root Mean Squared Error (RMSE), which measures both discrimination and calibration. It is the square root of the Mean Squared Error (MSE), which is also a valid metric in binary classification known as the Brier score [58]:

$$\text{MSE}\left(y,\mu \right)=\frac{1}{N}\sum_{i=1}^{N}{\left(y_{i}-E\left[\mu_{i}\right]\right)}^{2}$$

We also compare predicted prevalence and true prevalence in every region. Given a partition of field visits into *r* = 1, …, *T* geographic regions $R_{r}$, where $R_{1}\cup \ldots \cup R_{T}=\left[N\right]$ and $R_{r}\cap R_{s}=\emptyset \;\text{if}\;r\neq s$, we calculate the true prevalence in each region as the percent of field visits where the species occurs $p_{r}=\frac{1}{\|R_{r}\|}\sum_{i\in R_{r}}\;y_{i}$. We also calculate prediction intervals for the prevalence. This is done by sampling the species occurrence probabilities at every field visit location from their joint posterior distribution, then using these probabilities to sample the species occurrence (0/1). Repeating this sampling we obtain a distribution of predicted prevalences, where the 2.5% and 97.5% quantiles are used as the prediction intervals.

### 3.5 Simulation

**Fig 6:**
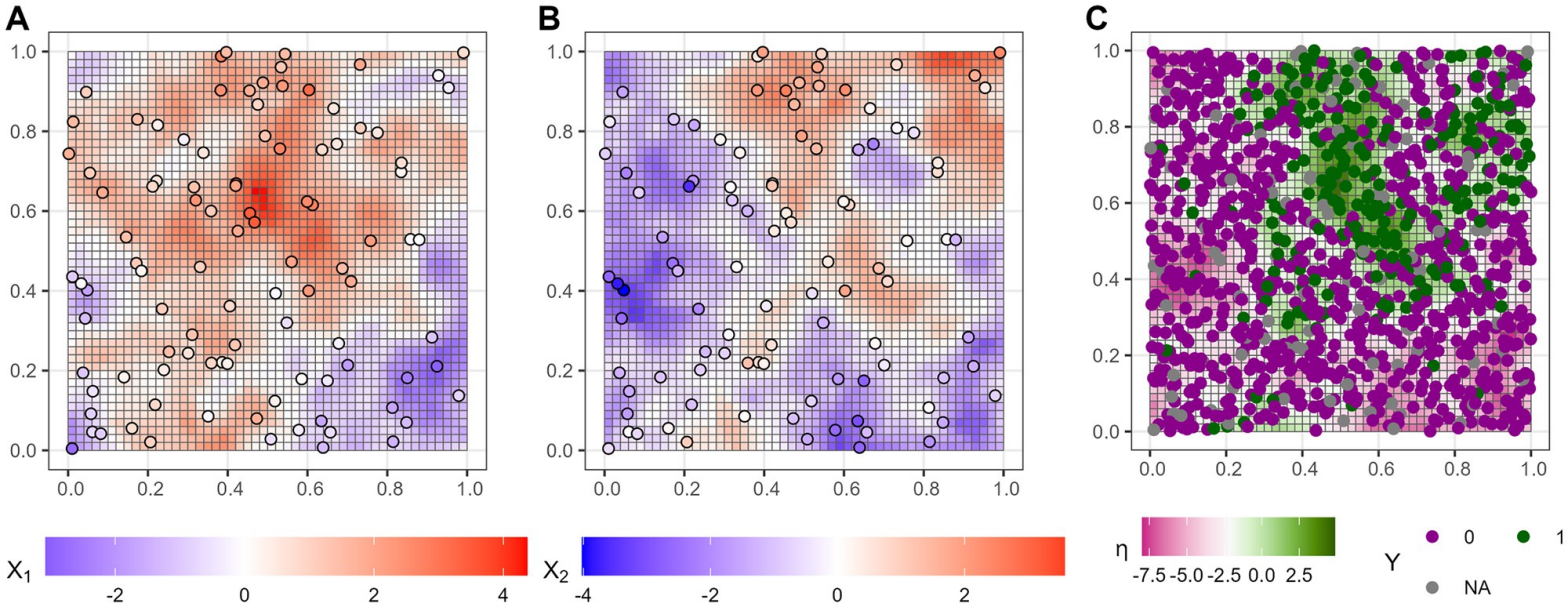
True abiotic covariate values *X*_1_(r), *X*_2_(r) have a latent gaussian field and the true species suitability $\eta =\beta_{0}+\beta_{1}^{†}X_{1}\left(r\right)+\beta_{2}^{†}X_{2}\left(r\right)+W\left(r\right)$ is defined by linear effects $\beta_{1}^{†},\beta_{2}^{†}$ and a residual spatial effect *W*(*r*). The random variables are defined on the entire unit square ***r*** (grid in A,B,C) but the observation locations of abiotics (dots in A,B) and species (dots in C) are chosen separately. This results in spatial misalignment, where the species occurrence *Y* is observed at many locations but not the abiotic measurement locations (NA).

It is not possible to know for sure which estimated associations are correct in a real data set since we do not know the ground truth. A simple simulation, illustrated in Fig 6, can be envisioned to assess which estimates are more correct. We sample data sets from an assumed parametric relationship defined by known parameter values, where a generative model of the data is given by the same likelihood that was used to define the models.

Let us define the simulation formally. Consider all locations on the unit square **r** = [0, 1] × [0, 1]. Suppose we have observations *Y*(***s***) of species occurrence at field visit locations ${\left\{s_{i}\right\}}_{i=1}^{N}\subset r$, and observations of abiotic variables $X_{1}^{*}\left(a\right)$ and $X_{2}^{*}\left(a\right)$ at measurement locations ${\left\{a_{i}\right\}}_{i=1}^{M}\subset r$. To mirror the scenario with our data set, we let *N* = 1000 and *M* = 100 to imply higher uncertainty about the abiotic values, and uniformly sample each location from the unit square. This results in spatial misalignment because the species and abiotic observations have different locations.

The abiotic observations are generated from latent variables that represent the true covariate values. We wish to define two covariates with spatial autocorrelation and confounding (Var[*X*_1_(***r***)] = Var[*X*_2_(***r***)] = 1.0 and Cov[*X*_1_(***r***), *X*_2_(***r***)] = 0.5). First, we define a latent Gaussian Random Field (GRF) on the unit square: let it have a variance parameter $\sigma^{2^{†}}=0.5$ and a range parameter *r*^†^ = 0.3. We then simulate three different realizations of the GRF: $Z(r),Z_{1}(r),Z_{2}(r)\sim \text{MVN}(0,\Sigma (\sigma^{2^{†}},r^{†}))$ according to the Matern covariance function. The true covariate values *X*_1_(***r***), *X*_2_(***r***) have the desired properties if we let *X*_1_(***r***) = *Z*(***r***) + *Z*_1_(***r***) and *X*_2_(***r***) = *Z*(***r***) + *Z*_2_(***r***). Their observed counterparts $X_{1}^{*}\left(r\right),X_{2}^{*}\left(r\right)$ are random variables with additional uncertainty caused by the measurement error, let it have a variance parameter $\delta^{2^{†}}=0.3$:

$$\begin{array}{c} \begin{array}{cc} X_{1}^{*}\left(r\right) & =X_{1}\left(r\right)+\epsilon \\ X_{2}^{*}\left(r\right) & =X_{2}\left(r\right)+\epsilon \\ \epsilon & ∼N(0,{\delta^{2}}^{†}) \end{array} \end{array}$$ (8)

The true species occurrence probability ***μ*** at every location is defined using the true covariates with linear effects, let them have values $\beta_{0}^{†}=-2,\beta_{1}^{†}=1,\beta_{2}^{†}=1$, and a spatial effect *W*(***r***):

$$\begin{array}{c} \begin{array}{cc} Y(r) & ∼\text{Bernoulli}(\mu )\\ \mu & =\frac{1}{1+e^{-\eta }}\\ \eta & =\beta_{0}+\beta_{1}^{†}X_{1}\left(r\right)+\beta_{2}^{†}X_{2}\left(r\right)+W\left(r\right)\\ W(r) & \;∼\text{MVN}(0,\Sigma \left({\sigma^{2}}^{†},r^{†}\right)) \end{array} \end{array}$$ (9)

Our simulation is therefore defined by the following ground truth parameter values: the number of locations (*N*, *M*), the GRF variance and range ($\sigma^{2^{†}}=0.5,r^{†}=0.3$), the measurement error variance ($\delta^{2^{†}}=0.3$), and the linear effects ($\beta_{0}^{†},\beta_{1}^{†},\beta_{2}^{†}$). In each run of the simulation *i*, we generate a dataset $D_{i}$ with a different realization of the abiotic covariates and species suitability using the ground truth parameter values, then sample the abiotic and species observation locations, and finally their observed values. This is illustrated in Fig 6 for one run of the simulation.

## 4 Results

In this section, we compare the interpretation and predictions given by the joint and two-stage models. First, we show all model output for a single species, including model parameters and predicted maps. We then compare all 50 species using summaries of these statistics and performance on the validation sets to assess model accuracy.

### 4.1 Example species: *Empetrum nigrum*

We chose to highlight *Empetrum nigrum* (Black crowberry) because it is one of the more common rare species and has clear soil type preferences. Moreover, the species is under threat of climate change in the Netherlands and could become extinct from the Netherlands with ongoing temperature raise. The species also has a longer lifespan, which makes it react to changes a bit slower than perennials but quicker than e.g. shrubs or trees. This species also shows differences in the model results that are representative of the differences that occurred in most species. *Empetrum nigrum* occurs in a minority of plots (2.4%), preferring dry, sunny to slightly shaded places with acidic sandy soil. Common occurrences in the Netherlands include the North Sea islands and large sandy nature areas.

**Fig 7:**
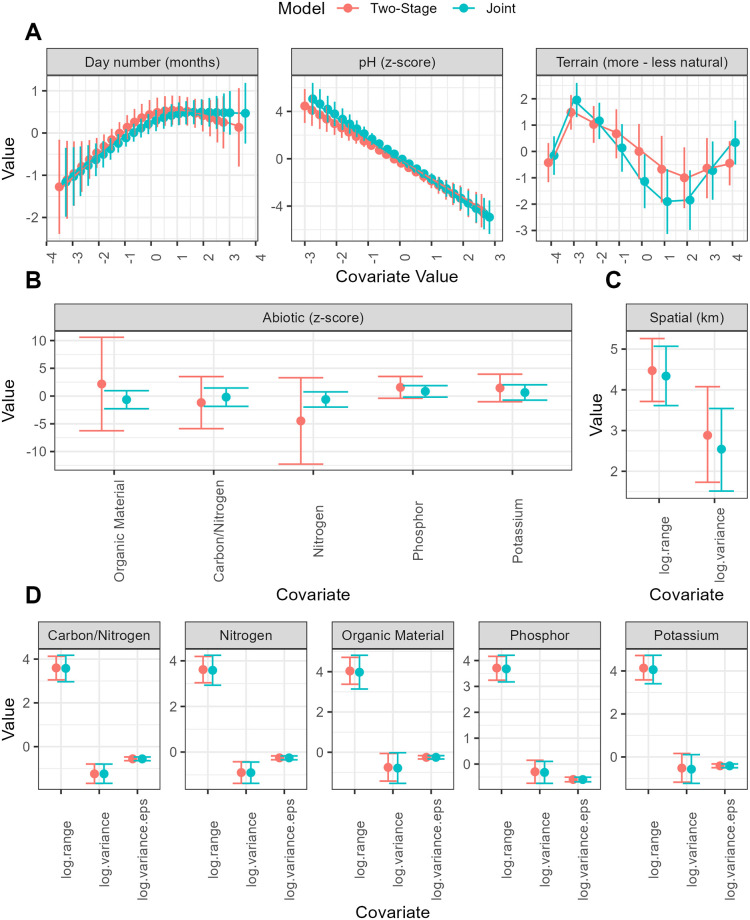
Comparison of the estimated parameters in the two-stage and joint models for the example species *Empetrum nigrum* (Crowberry). The first three panels (A) plot the SDM splines, the fourth panel (B) plots the coefficients for uncertain abiotic variables, the fifth panel (C) plots the parameters of the spatial component in the SDM, and the last five panels (D) plot the parameters of the spatial interpolation models of the abiotic variables and their measurement error.

We first show model parameters from both models in Fig 7. *Empetrum nigrum* has a strong tendency towards more acidic soils. The occurrence seems much more likely in natural areas, except those that are fully forested. For example, dry natural areas with high acidity have 8-fold increase in log-odds occurrence probability compared to forested areas with neutral pH. The joint model considers the day number, pH, and terrain to have a slightly stronger association with the species occurrence. However, there is a striking difference in the estimated associations and certainty of the five abiotic variables (Organic matter, Carbon/Nitrogen-Ratio, Nitrogen, Phosphorus, Potassium). The joint model that takes the uncertainty of the variables into account considers that associations are much smaller and more certain. For example, the association of Nitrogen and log-odds occurrence probability is estimated as -0.6 (std. 0.7) instead of -4.5 (std 4.0.) The estimates of the joint model are within credible intervals of the two-stage model. The spatial residual also tends to be slightly smaller in the joint model. Both models give almost identical estimates of the random field used to interpolate abiotic factors.

**Fig 8:**
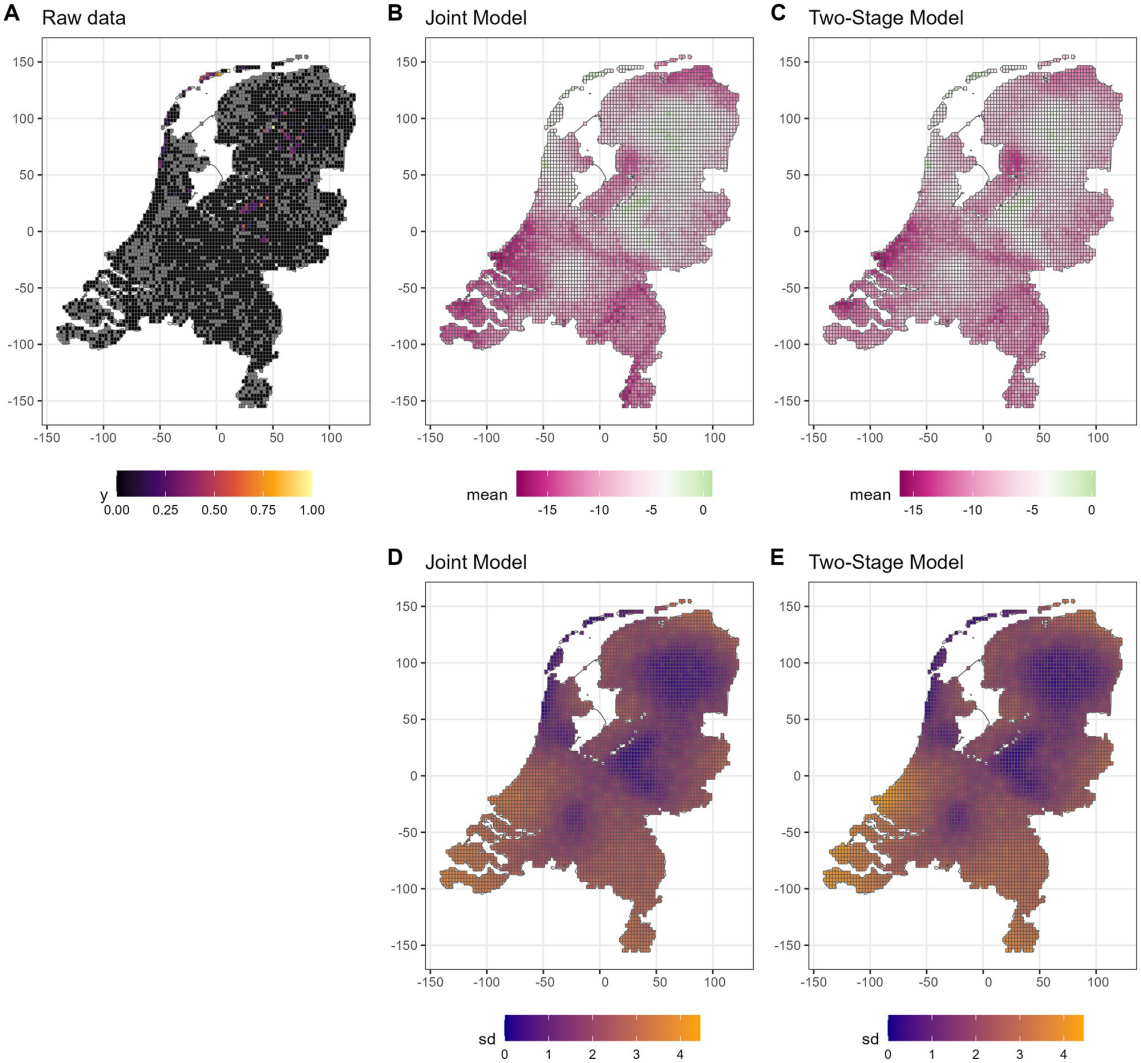
Left panel (A) is the observed species (*Empetrum nigrum*). The gray grid cells are where we had no observations at all, the black grid cells are where we did have observations, but not for these species. Ohter colors depict the fraction of how often this species was observed in plots in this cell. The top row middle (B) and right (C) panels compare the mean predicted log-odds occurrence probability in the joint and two-stage models. The bottom row middle (D) and right (E) panels compare the standard deviation, i.e. uncertainty, of predicted log-odds occurrence probability.

We provide all six predicted maps from both models: 1 species occurrence map (Fig 8) and 5 abiotic maps in the supplementary material for Organic matter (S4 Fig) Carbon/Nitrogen-Ratio (S5 Fig), Nitrogen (S6 Fig), Phosphorus (S7 Fig), Potassium (S8 Fig). We compare each map over all grid cells by contrasting the two model predictions using a scatter plot in S9 Fig. Predicted abiotic values in Netherlands are virtually identical (1.00 correlation) but the predicted species occurrence maps have small differences (0.94 correlation). The joint model considers that *Empetrum nigrum* has a slightly higher occurrence towards more suitable areas and lower occurrence in non-suitable areas based on the comparison of mean posterior log-odds occurrence probability in S9 Fig. The predicted occurrence map is also considered slightly more certain in the joint model because the standard deviation is below the diagonal.

### 4.2 All species

We have run both models over all 50 expert selected species. We summarize these results by first considering the differences in abiotic associations, then compare the predicted maps, and finally the predictive accuracy in cross-validated areas.

The relationship between species occurrence and Organic matter, Carbon/Nitrogen-Ratio, Nitrogen, Phosphorus, and Potassium is displayed in S10 Fig. The same trend applies over all species: the joint model produces estimates of association that tend to be smaller and are almost always more certain. These differences are strongest for relatively rarer species, for which it is difficult to confidently estimate the associations with species occurrence. For more common species, estimates are often very similar, see for example *Urtica dioica*, *Lolium perenne*, and *Quercus robur*. Even though most estimates are similar there are few differences, for example Nitrogen for the *Anthoxanthum odoratum*. We summarize the correlations of the estimated abiotic parameters over all 50 species in Table 2. The correlations are modest to strong (0.64–0.84), indicating that there are differences between the two models.

**Table 2:** Pearson correlation coefficient of the estimated abiotic associations in all 50 species between two-stage vs. joint model.

ORG_STOF.z	CN.z	N_totaal.z	P_totaal.z	K_CaCl2.z
0.62	0.71	0.83	0.84	0.78

**Fig 9:**
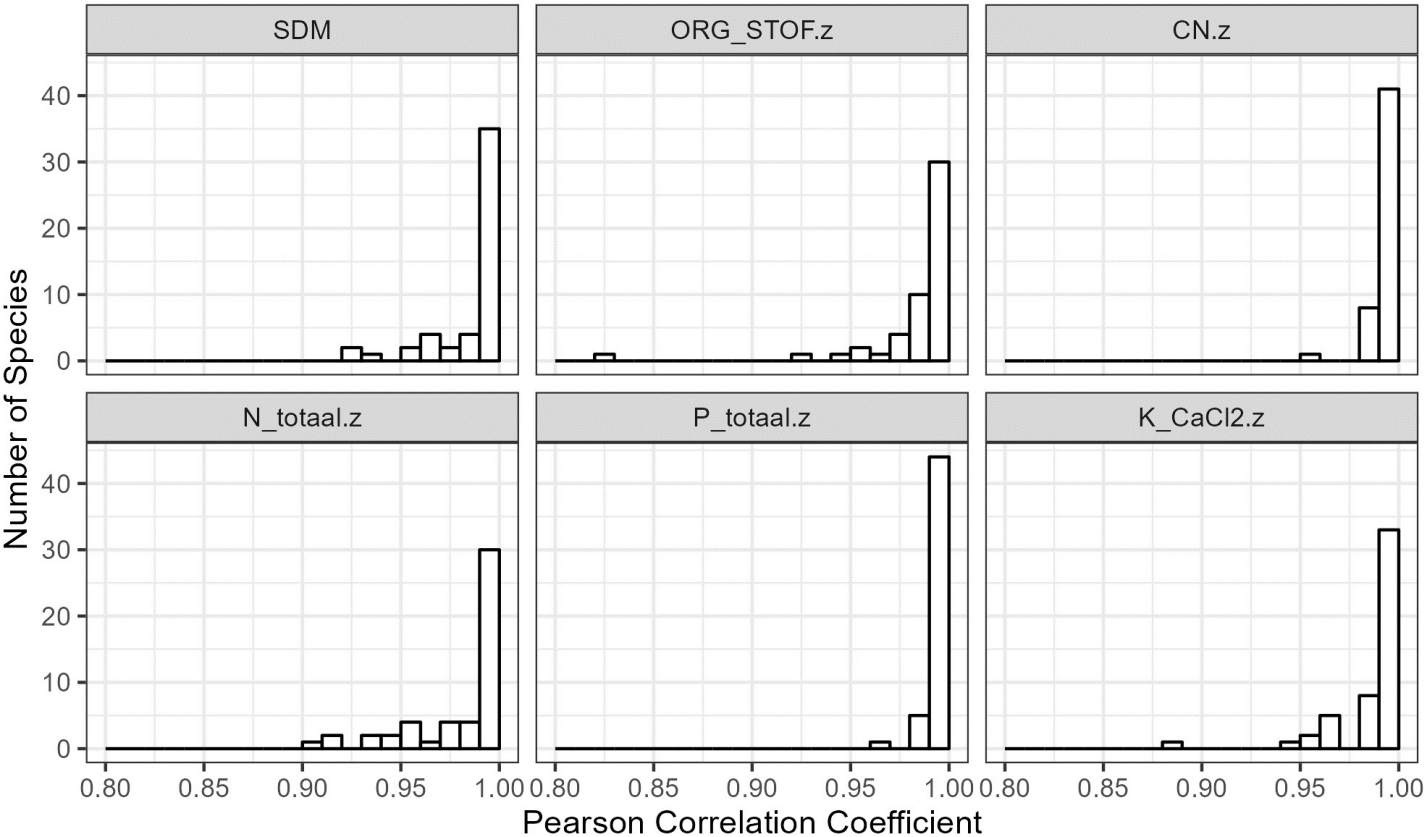
We compare how similar the 6 predicted maps appear by calculating Pearson correlation coefficients for 50 plant species. The maps are based on predicted mean log-odds occurrence probabilities (panel ‘SDM’) and predicted mean values (other panels). The histograms consist of 50 correlation coefficients calculated from the predicted grid cell values of the full-coverage map between the two-stage vs joint models.

We calculate a correlation coefficient from the predicted maps produced by the joint vs. two-stage model. Altogether, this results in 300 summaries of maps from 50 species and 6 predictions (SDM, ORG, C/N, N, P, K) displayed in S2 Table. The majority of maps estimated for different species are close to identical, but there are few exceptions with small differences that can occur in either estimated abiotic values or estimated species occurrence. For example, *Empetrum nigrum* has identical estimated abiotic values but shows different species occurrence maps, whereas *Achillea millefolium* has identical species occurrence and other abiotic values but shows a different estimated Nitrogen map. We visualize these summaries in Fig 9.

**Fig 10:**
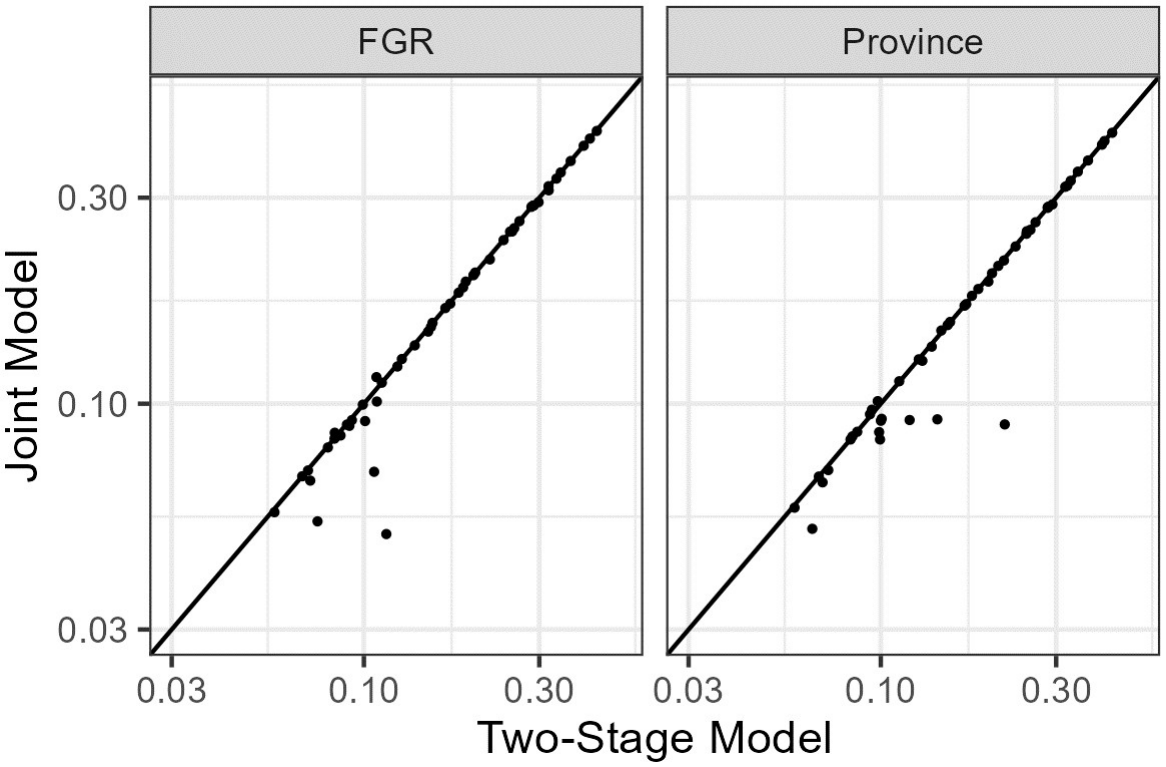
Root-mean-square error (RMSE) comparison of validation results between the joint and two-stage models. Dots represent 50 different plant species. The left panel plots leave-region-out validation, while the right panel plots leave-province-out validation. The joint model outperforms the two-stage model for species that are located below the diagonal.

We finally perform spatial cross-validation to compare out-of-sample species occurrence predictions to the true occurrence in each plot. The resulting RMSE for every species is summarized in Fig 10 and species specific RMSEs are listed in S3 Table. Generally, the joint model and the two-stage model display very similar RMSE. For some species the joint model appears more accurate; these include *Primula elatior*, *Asparagus officinalis*, *Puccinellia maritima*, *Dactylorhiza maculata*, and *Epipactis palustris*. The below diagonal area in Fig 10 signifies species for which the joint model has better performance.

We also predict the overall out-of-sample prevalence for all 50 species in each province and FGR. The models generally produce almost identical estimates of prevalence, with the joint model giving more confident estimates, i.e. narrower prediction intervals. The full results are given in S11 Fig for provinces and S12 Fig for FGRs. The confidence increases more for species that occur rarely in the data set, are present in only few areas, or are considered threatened. The number of times each species occurs is counted in S1 Table. For example, in many provinces and FGR regions the least observed species *Primula elatior* has 0–25% credible prevalence in the two-stage model but 0–5% credible prevalence in the joint model.

### 4.3 Simulation

**Fig 11:**
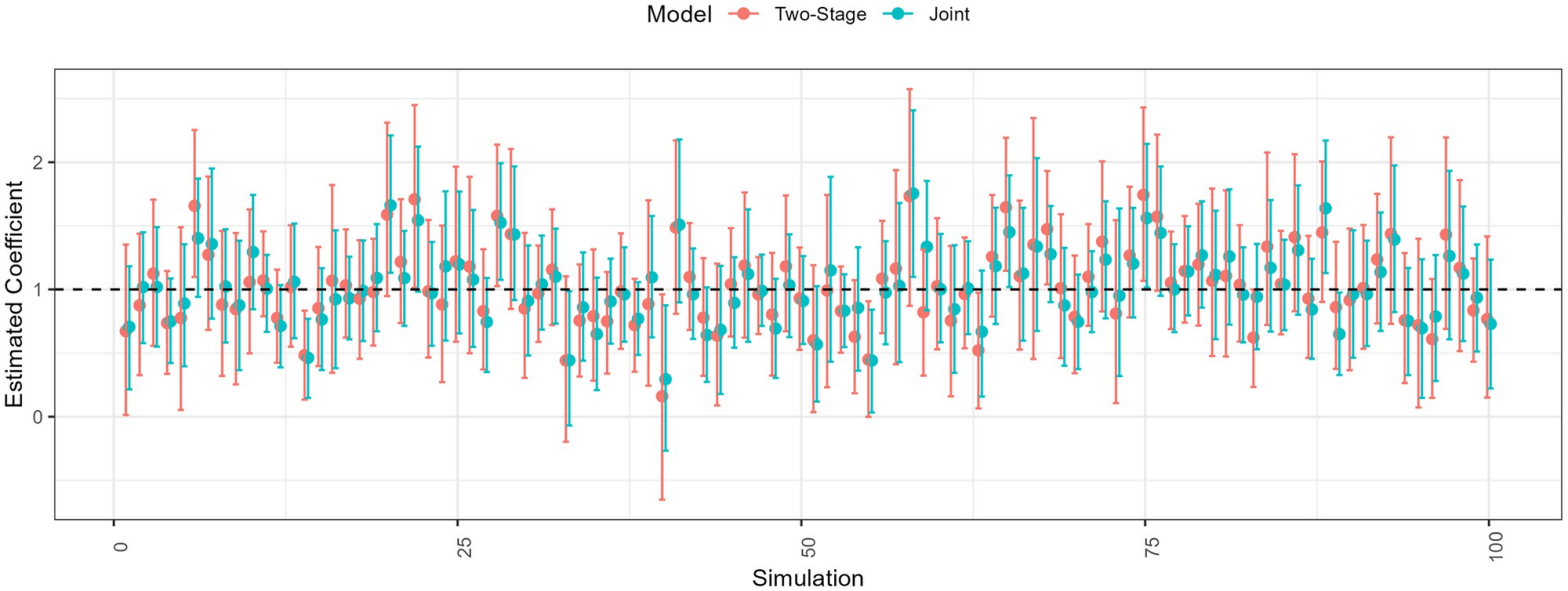
Estimates of the linear effect and its 95% credible interval in the data sets generated from the simulation with spatial misalignment and measurement error. The joint and two-stage model return estimates of the true value (dashed line). Both return unbiased estimates ($E_{D_{\rangle }}\left[E\left[\beta_{1}|D_{\rangle }\right]\right]\approx 1$) but the joint model is more certain ($E_{D_{\rangle }}\left[\sigma \left[\beta_{1}|D_{\rangle }\right]\right]$ 0.23 vs 0.28). The joint model estimates are generally more accurate (0.22 vs. 0.24 RMSE).

In the simulation, consider the covariate *X*_1_(***r***). It has a different random field in every run and a different estimated value $E[\beta_{1}|D_{i}]]$, even though the true effect $\beta_{1}^{†}$ stays the same. This enables assessing how well both models estimate the true parameter value in different data sets. We illustrate the estimates and their 95% credible interval from the simulations in Fig 11.

We can consider four simulation settings if we exclude either spatial misalignment or measurement error. In spatially aligned data, abiotic measurements have also been performed at the 1000 species observation locations (*M* = 1100). In simulation without measurement error, we set $\delta^{2^{†}}=0$. Kriging is not necessary if the data is spatially aligned because one may directly fit a regression model using the observed covariate values, which we call the ‘Direct’ model. A summary of all settings is displayed in S4 Table.

When there is no spatial misalignment or measurement error, all models return equally good estimates (1.01). Note that the direct model cannot be used to predict the map of abiotic values $E[X_{1}\left(r\right)]$ nor the map of species suitability E[Y(r)] because there is no kriging interpolation of abiotics to all grid points. When there is no spatial misalignment but observations suffer from measurement error, the Direct model suffers from an attenuated estimate (0.65) whereas the Two-Stage and Joint model work equally well (1.02). If there is spatial misalignment, the Joint and Two-Stage model return similar estimates (1.02 vs. 1.03) of the effect $E[\beta_{1}|D_{i}]$ but the Joint model has a lower estimated variance (0.23 vs 0.28). The Direct model cannot be fitted because the covariate values are unknown at species observation locations. The Joint model has a higher accuracy in the predicted species observations map (0.108 vs 0.110) and the predicted covariate map (0.565 vs. 0.625).

## 5 Discussion

### 5.1 Comparing the joint and two-stage models

We have compared two modelling approaches, where the two-stage modelling approach is simple but does not take into account the uncertainty in the abiotic variables. The joint modelling approach is based on more correct assumptions since there is uncertainty in the abiotic variables as evidenced from the kriging results. The joint model and two-stage model asymptotically give the same results if the uncertainty decreases with better sampling coverage and less measurement error. The model definitions become identical if there is no uncertainty in the abiotic variables used in the SDM. We found differences to occur often in the model interpretation, with the joint model delivering abiotic associations of smaller magnitude and more certainty. Differences in predictive accuracy to unseen areas also occur for some relatively rarer species. However, predicted maps of species occurrence and abiotic values that cover the Netherlands appear almost identical when trained with the full data set for most of the species.

In our case study there are two reasons why the two-stage model is probably sufficient for predictive purposes. First, the abiotic variables can be reliably estimated due to a long range of spatial correlation, with credible values 20–150 km, see Fig 7. Second, there are 12799 species observation locations as illustrated in Fig 1, thanks to the LMF data having a large sample size and stratification designed with the goal of obtaining reliable status of plant occurrence in the Netherlands.

In the simulation we could choose whether there is spatial misalignment or measurement error. The goal is to always use the true covariate values in the SDM, and how well they are recovered depends on both the observation uncertainty caused by the measurement error and the model uncertainty caused by the spatial misalignment. A regression model can be fitted directly if there is no spatial misalignment, but the measurement error caused bias when the observed values were used in place of the true values, which is known as ‘regression dilution’. Kriging in both the Two-Stage and Joint estimates the expected value at every location, i.e. value of the abiotics without measurement error, so the association seems to be fixed when these are used in the SDM. It seems like the kriging in the two-stage model can be sufficient for dealing with the measurement error, but the joint model can be beneficial for dealing with spatial misalignment. The accuracy of predicted maps was higher in the joint model, especially for the abiotic value, which makes sense because abiotics had significantly more spatial uncertainty than species occurrence in the simulation.

The context of the SDM modelling task is important. We investigated a large data set designed for plant monitoring. Several factors could contribute to more uncertainty in other models or case studies, including: smaller sample sizes of species or abiotic variables, more rare species, more complex models, more covariates, more variability in covariates, smaller spatial ranges of covariates, and more measurement error. With more uncertainty in the covariates the differences between the models could become more pronounced.

### 5.2 Related literature

Some studies in the SDM literature can be seen as predecessors of our approach. Many researchers have suggested that a Bayesian analysis would provide solutions to a range problems in SDMs, including uncertainty in the predictor variables. The same idea motivates the model presented in our paper, but we argue it allows for more realistic SDMs since we include multiple predictor variables, spatial autocorrelation in the response variable, uncertainty estimated from data, and both Berkson and Classical error.

The definition of Classical and Berkson errors is as follows [59]. Denote the true value of a covariate as the random variable *X* and the observed value as X*. Denote a new random variable L, Classical error *U*_C_ ∼ N(0, *δ*^2^) and Berkson error *U*_B_ ∼ N(0, *σ*^2^):

$$\begin{array}{c} \begin{array}{cc} X & =L+U_{B}\\ X^{*} & =L+U_{C} \end{array} \end{array}$$ (10)

This corresponds to Classical error when *U*_B_ = 0 since X* = *X* + *U*_C_, and Berkson error when *U*_C_ = 0 since *X* = X* + *U*_B_. It directly follows that observations with Classical error have more variance: Var[X*] = Var[*X*] + *δ*^2^, whereas observations with Berkson error have less variance: Var[*X*] = Var[X*] + *σ*^2^. If neither is zero, both types of error are present. The INLA package can easily support either type of error [60] or more complex schemes that include both errors with missing values in covariates [61].

**Fig 12:**
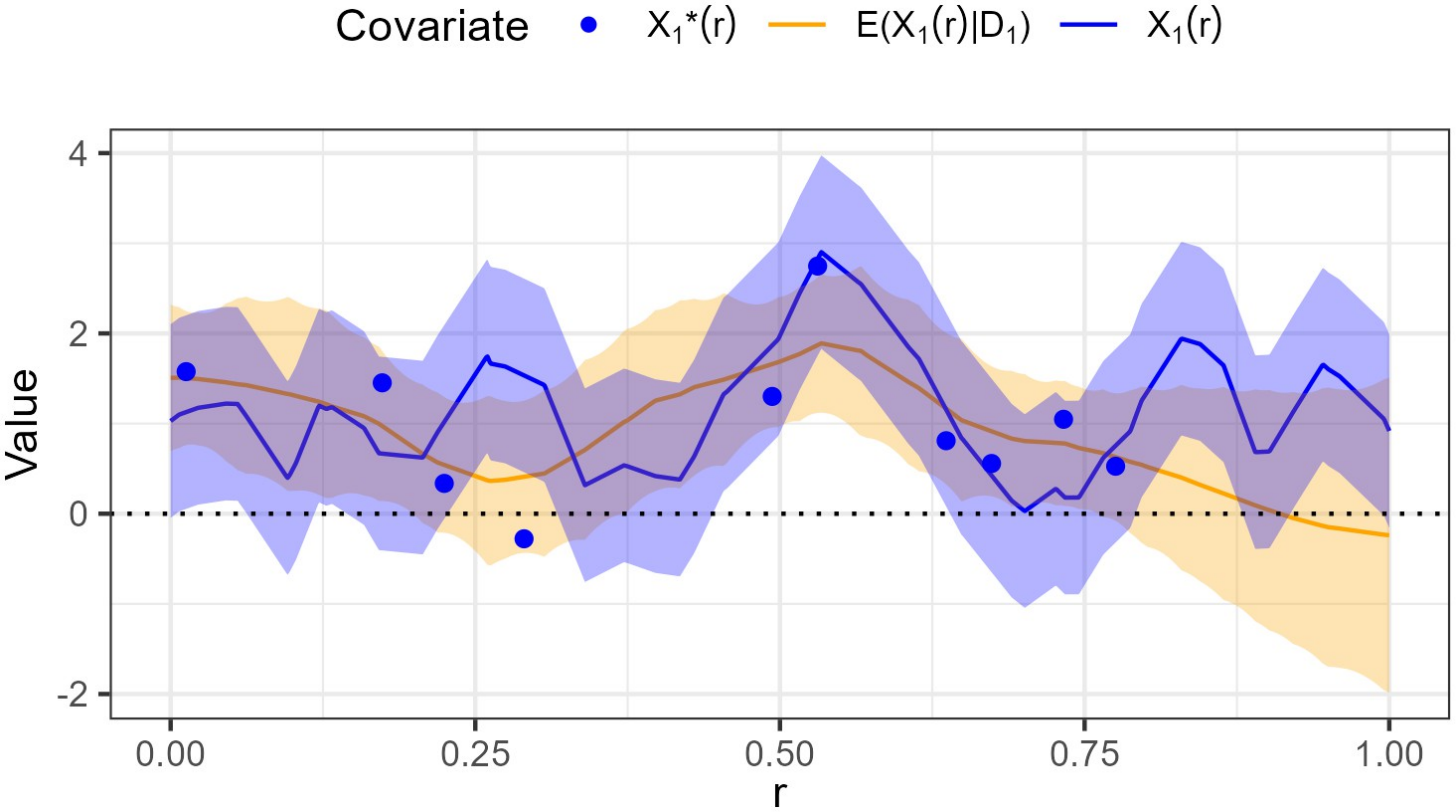
Example of the relationship between observed $X_{1}^{*}\left(r\right)$, kriging prediction $E[X_{1}\left(r\right)|D_{1}]]$, and true values *X*_1_(*r*) of the first covariate in the region *r* ∈ [0, 1] × 0.8 of the first simulation run. The observed value (blue dots) is a realization of the true value (blue line) with measurement error whose uncertainty is highlighted with 95% credible intervals (blue shaded). Kriging (orange line) estimates the true value whose uncertainty is highlighted with 95% credible intervals (orange shaded).

We connect the errors to our problem in Fig 12, which illustrates how uncertainty is created by kriging spatially misaligned values and having measurement error in the simulation. If repeated measurements of an abiotic factor give different results when measured at exactly the same location, we have measurement error. This corresponds to Classical error where the variance of the observations is larger than the variance of the true value. In Fig 12 for example, we have 4 measurements in the region *X* ∈ [0.63, 0.78], but we observe different outcomes due to the measurement error. If the observations of an abiotic factor are not done in all locations we use kriging to interpolate them. The kriging estimate is unbiased and the variance of predicted values is less than the variance of the true values. This corresponds to Berkson type error [62]. In Fig 12 for example, we have no measurements in the region *X* ∈ [0.78, 1.00], but we can estimate the abiotic values based on observations in the neighbouring regions. The predicted values are less variable than the true values. Regions with more observations imply less uncertainty; in the limit with unlimited observations we could perfectly recover the true value.

We summarize how our model can be seen as a generalization of previous studies in Table 3. Van Niel & Austin [28] explore the effects of Digital Elevation Model (DEM) error on SDMs, where an estimated error in the DEM is used to resample 10 different realizations of predictor variables. All of the steps of the modelling process were greatly affected by this type of error. The authors recommend that practitioners carefully consider how sensitive the modelling process and environmental variables are to errors in the source data. Denham et. al. [30] investigate a Bayesian framework for problems in ecology, where a classical measurement error model is inferred from validation data and linked to a regression model on these predictors. The results in real data yielded poor predictions, perhaps due to a faulty assumption of conditional independence between true and expert evaluated soil depth. Their simulations demonstrate the importance of using the measurement error model, instead of the standard model, for estimating the true relationship when the model assumptions are satisfied. McInerny & Purves [29] illustrate uncertainty in predictor variables using artificially generated data with a simple SDM where species absense or presense is determined by an unimodal response curve to temperature. They explain how uncertainty arising from fine-scale environmental variables causes ‘regression dilution’ and propose a correction using a Baysian method with a latent variable. Foster et. al. [27] investigate spatial misalignment in a synthetic survey where species presence or richness depends on the physical environment but the data sets are not co-located. They compare models fitted with true values, kriging predicted values, and Berkson error adjustment. However, even the true values resulted in bias, which the authors speculate could be due to spatial correlation. Stoklosa et. al. [31] show how classical error in predicted spatial climate variables can be incorporated into SDMs with hierarchical modelling and simulation-extrapolation (SIMEX). They perform a simple simulation with ground truth GLM parameters and fit the models to a species presence/absence data. Failing to account for error in variables resulted in biased parameter estimates and a loss of power as the error increases. Barber et al. [32] propose a hierarchical Bayes model to tackle the misalignment problem when species observations and annual mean temperature measurements have different locations. They compare 1) a joint model of the response variable and the covariate 2) a two-stage approach where the covariate is first predicted by kriging. They show that ignoring kriging uncertainty leads into different association, and interestingly the predicted map of species occurrence is more certain in the joint model.

**Table 3:** Summary of SDM literature most closely related to our research. Columns indicate: adjusting the models for uncertainty (Adjust), whether a GLM or GAM was used, multiple predictors (*X*_*j*_), Berkson error (*U*_B_), Classical error (*U*_C_), the predictors were estimated by kriging (*X*(***s***)), spatial component was included in the SDM (*W*(***s***)).

	Adjust	GLM	GAM	*X*_*j*_	*U*_B_	*U*_C_	*X*(*s*)	*W*(*s*)
Van Niel & Austin		X	X	X		X		
Denham et. al.	X	X		X		X		
McInerny & Purves	X		X		X			
Foster et. al.	X	X		X	X		X	
Stoklosa et. al.	X	X		X		X		
Barber et al.	X	X			X		X	X

### 5.3 Possible extensions

There are several improvements that can be considered in our work. First, the INLA R package has a technical limitation that uncertain covariates can only be included in linear terms, whereas it may be hypothesized that the abiotic variables ORG, C/N, N, P, K would have a unimodal response curve. We checked this assumption with a full GAMM where each covariate was modelled by a spline without uncertainty in covariates. However, results were often linear or at least monotonically increasing. There is no theoretical obstacle to implementing splines of uncertain covariates in a Bayesian framework. It may be interesting to investigate this with other, more general, packages such as Stan [63].

Second, computational efficiency will become a problem in increasingly complicated joint models. On average, to fit a single species takes 1 minute runtime for the two-stage model vs. 3 hours for the joint model, with identical components (4-core 2.5 Ghz, 8 GB RAM). A couple of hours is reasonable for a single modelling task and should not be an obstacle for taking the uncertainty into account, but performing the model fit over 50 species x 12+14 cross-validation splits would take months and necessitated a high performance cluster for parallel runs. The approximations used by INLA are quite fast, but more general approaches may result in an infeasible runtime. In practice, the benefits of taking uncertainty into account need to be balanced with the drawback of increasing computational burden.

Finally, future work might consider the uncertainty associated with time misalignment, i.e. situation where the field visit and abiotic measurement are done in different days or even years. INLA can support joint estimation of time series autocorrelation with spatial autocorrelation. This may be interesting especially for modelling effects of drought, acidification, climate change, and other environmental pressures over time.

## 6 Conclusions

We have investigated a new species distribution modelling approach where the uncertainty in abiotic variables caused by spatial misalignment and measurement error is taken into account. This is possible by fitting a joint model which simultaneously predicts the maps of abiotic values and species occurrence, feeding forward latent abiotic variables and their associated uncertainty estimated by kriging to the species distribution model. We have found that there are significant differences in the estimated associations between species occurrences and the uncertain abiotic variables. However, predicted maps for the Netherlands appear almost identical at this scale and resolution when models are fitted to the full data set. In testing out-of-sample predictive accuracy, we found that performance is very similar with the exception of few rare species where the new model is more accurate. It is interesting to note that information in the joint model goes both ways: abiotic variables will help to predict the species, but the species may also predict the abiotic variables. Our data set is a large nation-wide plant monitoring effort, and more uncertainty could imply larger differences between the models.

## Supporting information

S1 Table List of all 50 species chosen for the SDM. The table shows common names, Latin names, and the number of field visits where the species is found (n). Bold common names indicate that the species is on the Red List, indicating rare or threatened species in the Netherlands.
(PDF)

S2 Table Pearson correlation coefficients. We calculate a correlation coefficient between joint vs. two-stage model estimated maps for abiotic values and SDM log-odds occurrence probabilities in all 50 species.
(PDF)

S3 Table Validation RMSE in all 50 species of joint vs. two-stage model. (PDF)

S4 TableSummary of model estimates over all simulation runs.The first two columns signify spatial MisAlignment (MA) and Measurement Error (ME). Formally, the other columns are defined $E_{D}\left[E\left[\beta_{1}|D\right]\right]$, $E_{D}\left[\sigma \left[\beta_{1}|D\right]\right]$, $E_{D}\left[\text{RMSE}\left(\beta_{1}^{†},E_{D}\left[\beta_{1}|D\right]\right)\right]$, $E_{D}\left[\text{RMSE}\left(Y^{†}\left(r\right),E\left[Y\left(r\right)|D\right]\right)\right]$, $E_{D}\left[\text{RMSE}\left(X_{1}^{†}\left(r\right),E\left[X_{1}\left(r\right)|D\right]\right)\right]$ where the expectation $E_{D}$ is approximated by the sample mean over the sampled data sets $\left\{D_{1},\ldots D_{n}\right\}$ and ***r*** denotes the vector of all grid points.(PDF)

S1 Fig Interpolated pH map. The left panel (A) is the observed pH at abiotic measurement locations, the right panel (B) is the interpolated pH in every grid cell. Interpolation is based on spatial proximity, landuse, soiltype and *Calluna vulgaris* occurrence.
(TIF)

S2 Fig Landuse variable in every grid cell of the Netherlands. Natural areas are small and localized regions, whereas most of the country is agricultural or built terrain.
(TIF)

S3 Fig Landuse in every grid cell vs. plots. The number of field visits was stratified towards natural areas compared to the landuse in the entire country counted from grid cells.
(TIF)

S4 Fig Left panel (A) is the observed organic matter mean value in each grid cell. The top row middle (B) and right (C) panels compare the mean predicted value in the joint and two-stage models. The bottom row middle (D) and right (E) panels compare the standard deviation, i.e. uncertainty, of the predicted value. Joint model uses *Empetrum nigrum* as the SDM species.
(TIF)

S5 Fig Left panel (A) is the observed C/N-ratio mean value in each grid cell. The top row middle (B) and right (C) panels compare the mean predicted value in the joint and two-stage models. The bottom row middle (D) and right (E) panels compare the standard deviation, i.e. uncertainty, of the predicted value. Joint model uses *Empetrum nigrum* as the SDM species.
(TIF)

S6 Fig Left panel (A) is the observed total nitrogen mean value in each grid cell. The top row middle (B) and right (C) panels compare the mean predicted value in the joint and two-stage models. The bottom row middle (D) and right (E) panels compare the standard deviation, i.e. uncertainty, of the predicted value. Joint model uses *Empetrum nigrum* as the SDM species.
(TIF)

S7 Fig Left panel (A) is the observed total phosphorus mean value in each grid cell. The top row middle (B) and right (C) panels compare the mean predicted value in the joint and two-stage models. The bottom row middle (D) and right (E) panels compare the standard deviation, i.e. uncertainty, of the predicted value. Joint model uses *Empetrum nigrum* as the SDM species.
(TIF)

S8 Fig Left panel (A) is the observed total potassium mean value in each grid cell. The top row middle (B) and right (C) panels compare the mean predicted value in the joint and two-stage models. The bottom row middle (D) and right (E) panels compare the standard deviation, i.e. uncertainty, of the predicted value. Joint model uses *Empetrum nigrum* as the SDM species.
(TIF)

S9 Fig Example species *Empetrum nigrum* (Crowberry) scatter plots that compare, at every grid cell, the predicted log-odds for the SDM and the predicted values for abiotic variables. The top 6 panels illustrate the mean predicted value, while the bottom 6 panels present the standard deviation of the predicted value, i.e. uncertainty of the prediction.
(TIF)

S10 Fig Every panel plots for the given species the estimated associations between species occurrence and uncertain abiotic variables in the two-stage and joint model. (TIF)

S11 Fig Every panel plots for the given species the predicted prevalence in the two-stage and joint model using leave-province-out validation. These predictions are compared with the true prevalence (green) in each province.
(TIF)

S12 Fig Every panel plots for the given species the predicted prevalence in the two-stage and joint model using leave-region-out validation. These predictions are compared with the true prevalence (green) in each FGR region.
(TIF)
