# Supplementary material for: A joint model for the estimation of species distributions and environmental characteristics from point-referenced data
Source: PLoS One. 2024 Jun 21;19(6):e0304942. doi: 10.1371/journal.pone.0304942 (PMC11192322; doi:10.1371/journal.pone.0304942)
Supplement: S1 Table — The table shows common names, Latin names, and the number of field visits where the species is found (n). Bold common names indicate that the species is on the Red List, indicating rare or threatened species in the Netherlands. (PDF) [file pone.0304942.s001.pdf]

S1 Table. List of all 50 species chosen for the SDM The table shows common names, Latin names, and the number of field visits where the species is found (n). Bold common names indicate that the species is on the Red List, indicating rare or threatened species in the Netherlands.

**Common name**	**Latin name**	**n**
Yorkshire-fog	*Holcus lanatus*	4104
Pedunculate Oak	*Quercus robur*	3938
Common Nettle	*Urtica dioica*	3137
Soft-rush	*Juncus effusus*	2284
Broad Buckler-fern	*Dryopteris dilatata*	2080
Sweet Vernal-grass	*Anthoxanthum odoratum*	1882
Downy Birch	*Betula pubescens*	1844
Deschampsia flexuosa	*Deschampsia flexuosa*	1785
Silver Birch	*Betula pendula*	1566
Perennial Rye-grass	*Lolium perenne*	1434
Narrow Buckler-fern	*Dryopteris carthusiana*	1297
Water Mint	*Mentha aquatica*	1199
Ash	*Fraxinus excelsior*	1171
Cross-leaved Heath	*Erica tetralix*	1015
Holly	*Ilex aquifolium*	958
Common Ragwort	*Jacobaea vulgaris*	955
Yarrow	*Achillea millefolium*	915
Northern red oak	*Quercus rubra*	702
Common Hazel	*Corylus avellana*	620
Bird Cherry	*Prunus padus*	580
Creeping Willow	*Salix repens*	528
Brown Sedge	*Carex disticha*	469
Common Cottongrass	*Eriophorum angustifolium*	432
Field Maple	*Acer campestre*	428
Brown Bent	*Agrostis vinealis*	387
Common Carrot	*Daucus carota*	304
Crowberry	*Empetrum nigrum*	302
Tufted Hair-grass	*Deschampsia cespitosa*	288
Aspen	*Populus tremula*	238
Wild Cherry	*Prunus avium*	209
Bulbous Buttercup	*Ranunculus bulbosus*	195
**Oblong-leaved Sundew**	*Drosera intermedia*	169
Wood Anemone	*Anemone nemorosa*	130
Biting Stonecrop	*Sedum acre*	111
**Bog-myrtle**	*Myrica gale*	105
**Marsh Helleborine**	*Epipactis palustris*	102
**Heath Spotted-orchid**	*Dactylorhiza maculata*	101
Broad-leaved Helleborine	*Epipactis helleborine*	96
May Lily	*Maianthemum bifolium*	91
Wood-sorrel	*Oxalis acetosella*	90
**Grass-of-Parnassus**	*Parnassia palustris*	87
**Hare's-tail Cottongrass**	*Eriophorum vaginatum*	81
**Yellow-rattle**	*Rhinanthus minor*	77
**Wild Strawberry**	*Fragaria vesca*	63
Slender Rush	*Juncus tenuis*	59

**Common name**	**Latin name**	**n**
Small cranberry	*Vaccinium oxycoccos*	57
**Quaking-grass**	*Briza media*	40
Common Saltmarsh-grass	*Puccinellia maritima*	37
Edible asparagus	*Asparagus officinalis*	31
Oxlip	*Primula elatior*	24
