# Supplementary material for: A joint model for the estimation of species distributions and environmental characteristics from point-referenced data
Source: PLoS One. 2024 Jun 21;19(6):e0304942. doi: 10.1371/journal.pone.0304942 (PMC11192322; doi:10.1371/journal.pone.0304942)
Supplement: S2 Table — We calculate a correlation coefficient between joint vs. two-stage model estimated maps for abiotic values and SDM log-odds occurrence probabilities in all 50 species. (PDF) [file pone.0304942.s002.pdf]

S2 Table. Pearson correlation coefficients. We calculate a correlation coefficient between joint vs. two-stage model estimated maps for abiotic values and SDM log-odds occurrence probabilities in all 50 species.

species	ORG_STOF.z	CN.z	N.z	P.z	K.z	SDM
Acer campestre	1.00	1.00	0.99	0.99	0.98	1.00
Achillea millefolium	1.00	1.00	0.95	1.00	1.00	1.00
Agrostis vinealis	0.99	1.00	0.98	1.00	1.00	0.99
Anemone nemorosa	1.00	0.99	0.99	0.99	0.99	0.96
Anthoxanthum odoratum	0.97	1.00	0.91	0.99	0.97	0.99
Asparagus officinalis	0.98	0.98	1.00	0.99	0.99	0.92
Betula pendula	1.00	1.00	0.98	0.99	1.00	1.00
Betula pubescens	1.00	1.00	0.99	1.00	0.98	1.00
Briza media	1.00	1.00	1.00	1.00	1.00	0.96
Carex disticha	1.00	0.99	0.94	0.99	0.99	1.00
Corylus avellana	1.00	0.99	1.00	1.00	0.96	1.00
Dactylorhiza maculata	1.00	1.00	0.99	1.00	0.97	0.93
Daucus carota	1.00	1.00	0.99	1.00	0.98	1.00
Deschampsia cespitosa	0.99	0.99	1.00	0.99	0.96	1.00
Deschampsia flexuosa	0.99	1.00	0.97	0.99	0.98	0.99
Drosera intermedia	1.00	0.99	1.00	1.00	1.00	1.00
Dryopteris carthusiana	0.99	1.00	0.97	1.00	0.95	1.00
Dryopteris dilatata	0.99	0.98	0.95	0.99	0.95	1.00
Empetrum nigrum	1.00	1.00	1.00	1.00	1.00	0.94
Epipactis helleborine	0.99	1.00	0.99	1.00	0.96	0.99
Epipactis palustris	0.98	0.99	1.00	1.00	1.00	0.99
Erica tetralix	1.00	0.99	1.00	1.00	0.99	1.00
Eriophorum angustifolium	0.99	0.96	0.99	1.00	0.99	0.99
Eriophorum vaginatum	1.00	1.00	0.99	1.00	1.00	0.95
Fragaria vesca	0.98	0.99	0.99	1.00	0.99	0.99
Fraxinus excelsior	0.96	1.00	1.00	1.00	1.00	1.00
Holcus lanatus	0.99	1.00	0.94	0.96	1.00	1.00
Ilex aquifolium	0.95	0.99	0.96	1.00	1.00	1.00
Jacobaea vulgaris	0.82	0.99	0.97	1.00	1.00	0.99
Juncus effusus	1.00	1.00	0.91	0.99	0.88	1.00
Juncus tenuis	1.00	0.99	0.99	1.00	1.00	0.99
Lolium perenne	0.94	0.98	0.94	1.00	0.99	1.00
Maianthemum bifolium	1.00	1.00	1.00	1.00	0.99	0.96
Mentha aquatica	0.99	1.00	0.97	1.00	1.00	1.00
Myrica gale	1.00	1.00	1.00	1.00	0.99	0.97
Oxalis acetosella	1.00	1.00	1.00	0.99	1.00	0.99
Parnassia palustris	0.98	1.00	1.00	1.00	1.00	0.98
Populus tremula	0.99	1.00	1.00	1.00	1.00	1.00
Primula elatior	1.00	1.00	1.00	1.00	1.00	0.97
Prunus avium	1.00	1.00	0.98	0.99	0.99	0.99
Prunus padus	0.99	1.00	1.00	1.00	0.99	1.00
Puccinellia maritima	1.00	1.00	1.00	1.00	0.99	0.96
Quercus robur	0.99	0.99	0.92	1.00	1.00	1.00
Quercus rubra	0.99	0.99	0.96	1.00	1.00	1.00
Ranunculus bulbosus	0.99	0.99	0.96	1.00	1.00	0.99
Rhinanthus minor	0.99	1.00	0.99	1.00	0.99	1.00

species	ORG_STOF.z	CN.z	N.z	P.z	K.z	SDM
Salix repens	0.97	1.00	1.00	0.99	1.00	1.00
Sedum acre	0.93	0.99	0.99	1.00	1.00	0.97
Urtica dioica	0.98	1.00	1.00	0.98	0.95	1.00
Vaccinium oxycoccos	1.00	1.00	1.00	1.00	0.99	0.99
