# Supplementary material for: A joint model for the estimation of species distributions and environmental characteristics from point-referenced data
Source: PLoS One. 2024 Jun 21;19(6):e0304942. doi: 10.1371/journal.pone.0304942 (PMC11192322; doi:10.1371/journal.pone.0304942)
Supplement: S3 Table — (PDF) [file pone.0304942.s003.pdf]

S3 Table. Validation RMSE in all 50 species of joint vs. two-stage model.

Species	Province Two-Stage	Province Joint	FGR Two-Stage	FGR Joint
Acer campestre	0.169	0.169	0.167	0.167
Achillea millefolium	0.250	0.251	0.250	0.250
Agrostis vinealis	0.171	0.170	0.172	0.171
Anemone nemorosa	0.093	0.095	0.099	0.100
Anthoxanthum odoratum	0.329	0.328	0.334	0.332
Asparagus officinalis	0.065	0.051	0.115	0.050
Betula pendula	0.322	0.320	0.318	0.312
Betula pubescens	0.344	0.345	0.343	0.343
Briza media	0.058	0.057	0.057	0.056
Carex disticha	0.184	0.184	0.186	0.186
Corylus avellana	0.209	0.209	0.201	0.201
Dactylorhiza maculata	0.217	0.090	0.091	0.089
Daucus carota	0.154	0.155	0.154	0.154
Deschampsia cespitosa	0.146	0.148	0.150	0.147
Deschampsia flexuosa	0.285	0.285	0.299	0.293
Drosera intermedia	0.112	0.113	0.112	0.112
Dryopteris carthusiana	0.286	0.286	0.288	0.288
Dryopteris dilatata	0.318	0.318	0.318	0.319
Empetrum nigrum	0.152	0.152	0.152	0.151
Epipactis helleborine	0.086	0.086	0.108	0.101
Epipactis palustris	0.120	0.092	0.090	0.089
Erica tetralix	0.233	0.231	0.240	0.239
Eriophorum angustifolium	0.177	0.178	0.181	0.181
Eriophorum vaginatum	0.098	0.101	0.101	0.091
Fragaria vesca	0.072	0.070	0.070	0.070
Fraxinus excelsior	0.264	0.263	0.265	0.265
Holcus lanatus	0.426	0.424	0.430	0.428
Ilex aquifolium	0.249	0.248	0.253	0.251
Jacobaea vulgaris	0.255	0.253	0.256	0.255
Juncus effusus	0.366	0.366	0.365	0.365
Juncus tenuis	0.068	0.068	0.068	0.068
Lolium perenne	0.293	0.289	0.290	0.288
Maianthemum bifolium	0.083	0.083	0.083	0.083
Mentha aquatica	0.284	0.284	0.286	0.286
Myrica gale	0.100	0.091	0.108	0.115
Oxalis acetosella	0.084	0.084	0.083	0.086
Parnassia palustris	0.101	0.092	0.086	0.084
Populus tremula	0.138	0.136	0.138	0.136
Primula elatior	0.099	0.086	0.075	0.053
Prunus avium	0.127	0.127	0.127	0.127
Prunus padus	0.201	0.201	0.199	0.198
Puccinellia maritima	0.142	0.092	0.107	0.070
Quercus robur	0.406	0.406	0.412	0.411
Quercus rubra	0.216	0.215	0.220	0.216
Ranunculus bulbosus	0.130	0.126	0.123	0.122
Rhinanthus minor	0.100	0.083	0.080	0.079

S3 Table. Validation RMSE in all 50 species of joint vs. two-stage model.

Species	Province Two-Stage	Province Joint	FGR Two-Stage	FGR Joint
Salix repens	0.196	0.192	0.189	0.192
Sedum acre	0.095	0.097	0.093	0.092
Urtica dioica	0.400	0.398	0.396	0.396
Vaccinium oxycoccos	0.069	0.066	0.071	0.066
