# Supplementary material for: A joint model for the estimation of species distributions and environmental characteristics from point-referenced data
Source: PLoS One. 2024 Jun 21;19(6):e0304942. doi: 10.1371/journal.pone.0304942 (PMC11192322; doi:10.1371/journal.pone.0304942)
Supplement: S4 Table — The first two columns signify spatial MisAlignment (MA) and Measurement Error (ME). Formally, the other columns are defined ED[E[β1|D]], ED[σ[β1|D]], ED[RMSE(β1†,ED[β1|D])], ED[RMSE(Y†(r),E[Y(r)|D])], ED[RMSE(X1†(r),E[X1(r)|D])] where the expectation ED is approximated by the sample mean over the sampled data sets {D1,…Dn} and r denotes the vector of all grid points. (PDF) [file pone.0304942.s004.pdf]

S4 Table. Summary of model estimates over all simulation runs. The first two columns signify spatial MisAlignment (MA) and Measurement Error (ME). Formally, the other columns are defined $\mathbb{E}_{\mathcal{D}}[\mathbb{E}[\beta_1|\mathcal{D}]]$, $\mathbb{E}_{\mathcal{D}}[\sigma[\beta_1|\mathcal{D}]]$, $\mathbb{E}_{\mathcal{D}}[\text{RMSE}(\beta_1^{\dagger}, \mathbb{E}_{\mathcal{D}}[\beta_1|\mathcal{D}])]$, $\mathbb{E}_{\mathcal{D}}[\text{RMSE}(Y^{\dagger}(\boldsymbol{r}), \mathbb{E}[Y(\boldsymbol{r})|\mathcal{D}])]$, $\mathbb{E}_{\mathcal{D}}[\text{RMSE}(X_1^{\dagger}(\boldsymbol{r}), \mathbb{E}[X_1(\boldsymbol{r})|\mathcal{D}])]$ where the expectation $\mathbb{E}_{\mathcal{D}}$ is approximated by the sample mean over the sampled data sets $\{\mathcal{D}_1, \ldots \mathcal{D}_n\}$ and $\boldsymbol{r}$ denotes the vector of all grid points.

**MA**	**ME**	**Model**	**Mean** $\mathbb{E}[\beta_1]$	**Mean** $\sigma[\beta_1]$	**RMSE** $\mathbb{E}[\beta_1]$	**RMSE** $\mathbb{E}[Y(\boldsymbol{r})]$	**RMSE** $\mathbb{E}[X_1(\boldsymbol{r})]$
		Direct	1.01	0.17	0.14		
		Two-Stage	1.01	0.17	0.14	0.073	0.039
		Joint	1.01	0.17	0.15	0.074	0.039
	x	Direct	0.65	0.12	0.35		
	x	Two-Stage	1.02	0.18	0.16	0.090	0.282
	x	Joint	1.02	0.18	0.16	0.089	0.280
x		Direct					
x		Two-Stage	0.99	0.24	0.20	0.106	0.489
x		Joint	0.99	0.20	0.20	0.104	0.445
x	x	Direct					
x	x	Two-Stage	1.03	0.28	0.24	0.110	0.625
x	x	Joint	1.02	0.23	0.22	0.108	0.565
