# Supplementary figures and images for: A joint model for the estimation of species distributions and environmental characteristics from point-referenced data
Source: PLoS One. 2024 Jun 21;19(6):e0304942. doi: 10.1371/journal.pone.0304942 (PMC11192322; doi:10.1371/journal.pone.0304942)

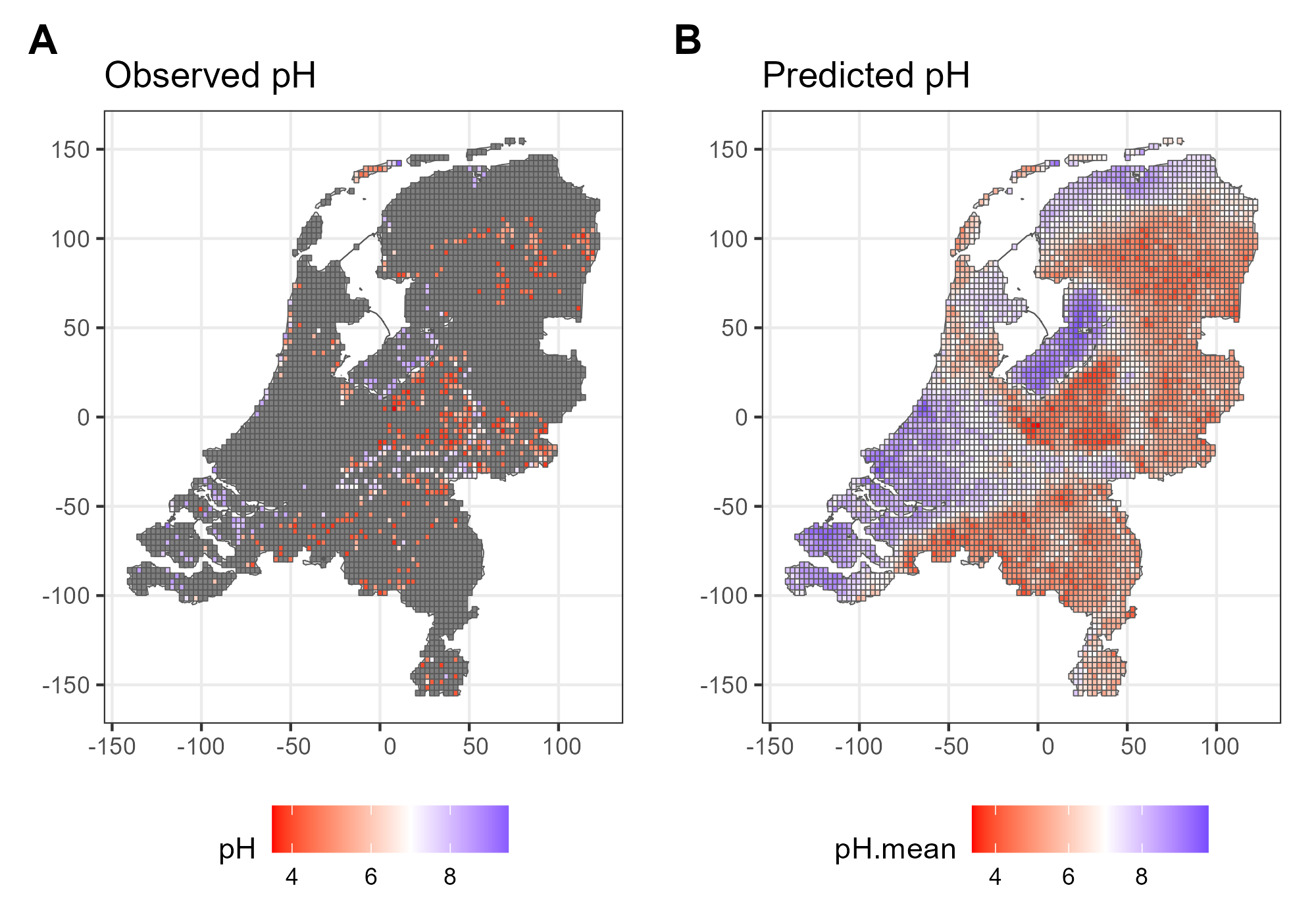

Supplement: S1 Fig — The left panel (A) is the observed pH at abiotic measurement locations, the right panel (B) is the interpolated pH in every grid cell. Interpolation is based on spatial proximity, landuse, soiltype and Calluna vulgaris occurrence. (TIF) [file pone.0304942.s005.tif]

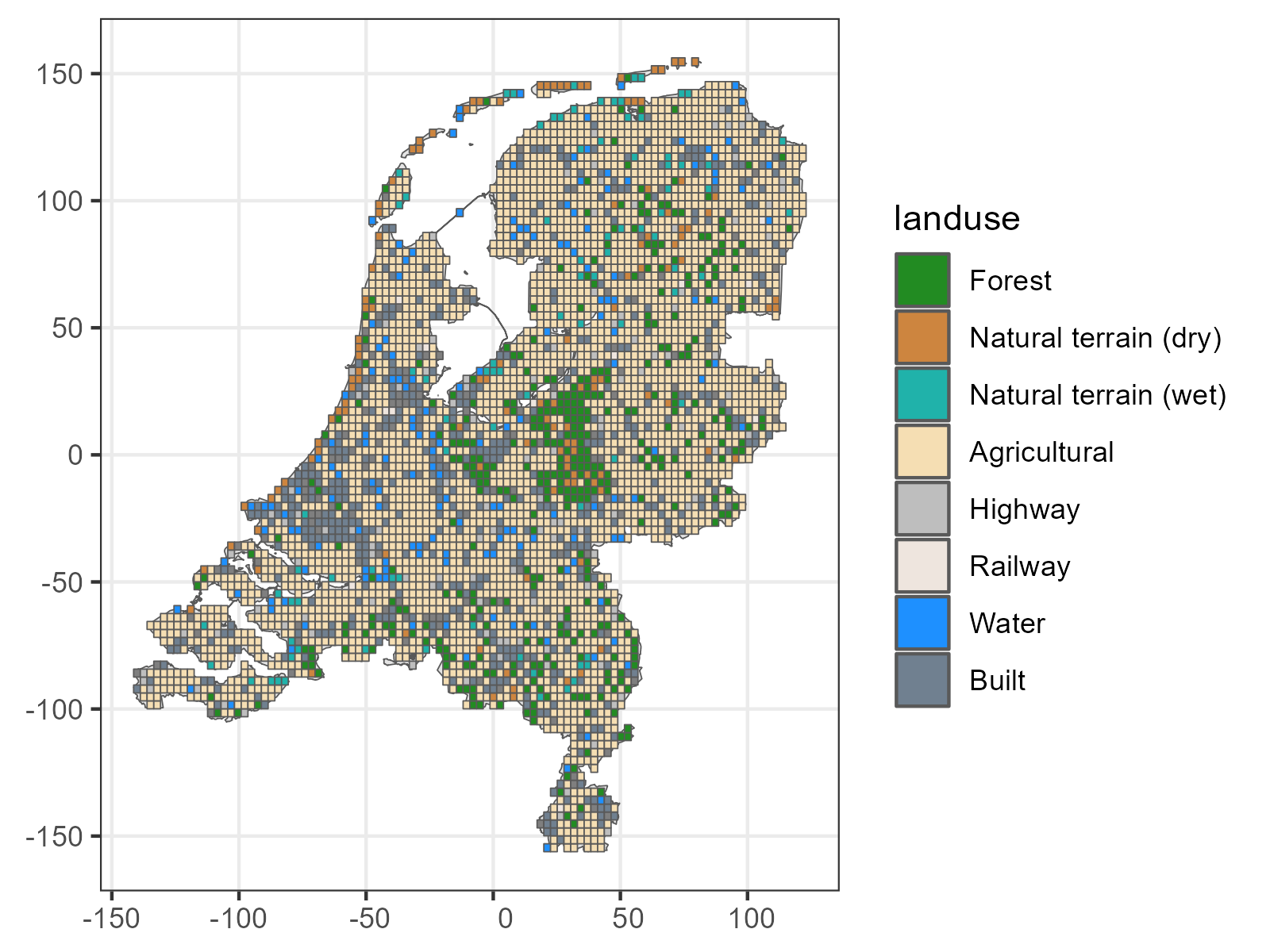

Supplement: S2 Fig — Natural areas are small and localized regions, whereas most of the country is agricultural or built terrain. (TIF) [file pone.0304942.s006.tif]

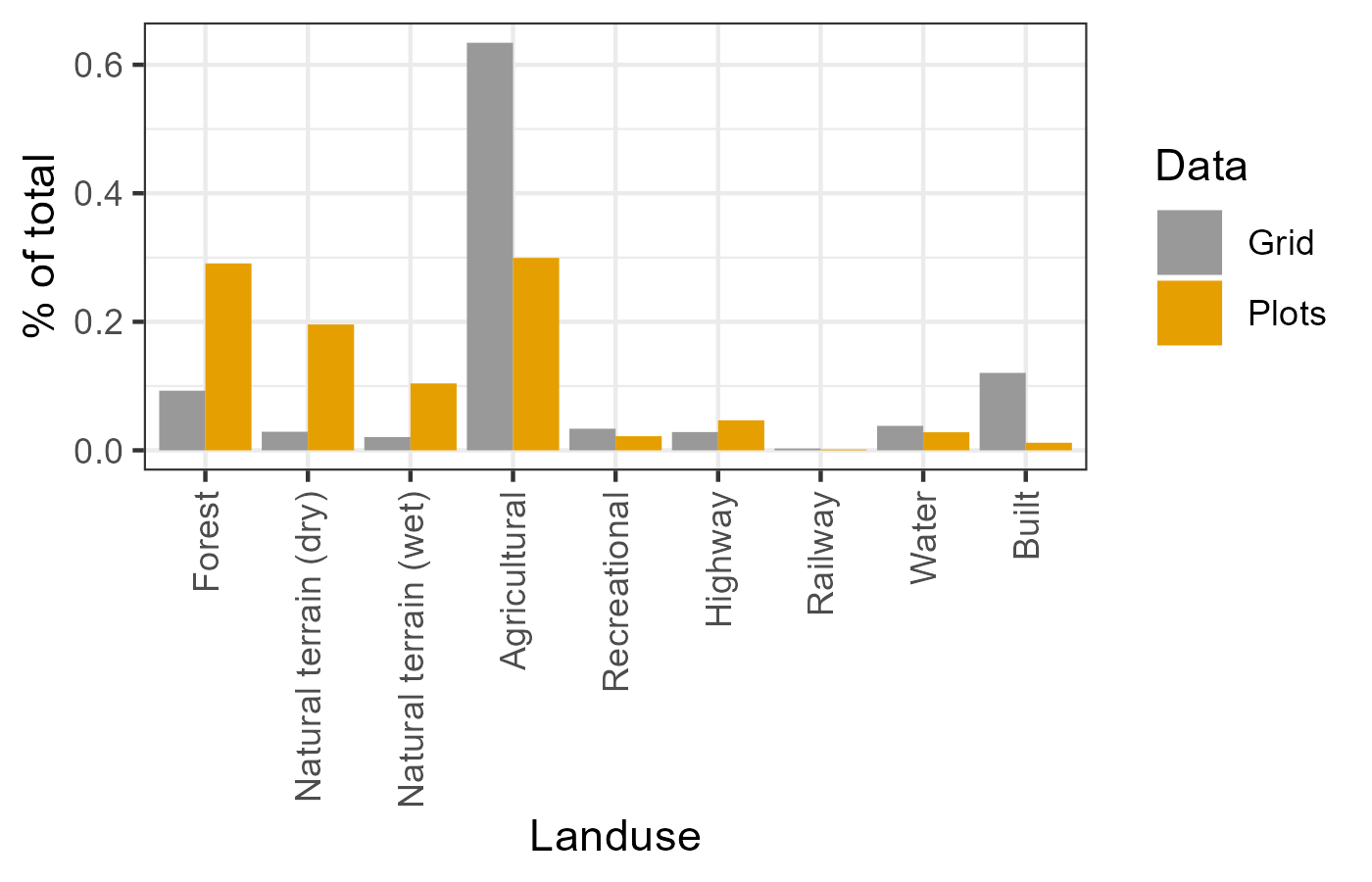

Supplement: S3 Fig — The number of field visits was stratified towards natural areas compared to the landuse in the entire country counted from grid cells. (TIF) [file pone.0304942.s007.tif]

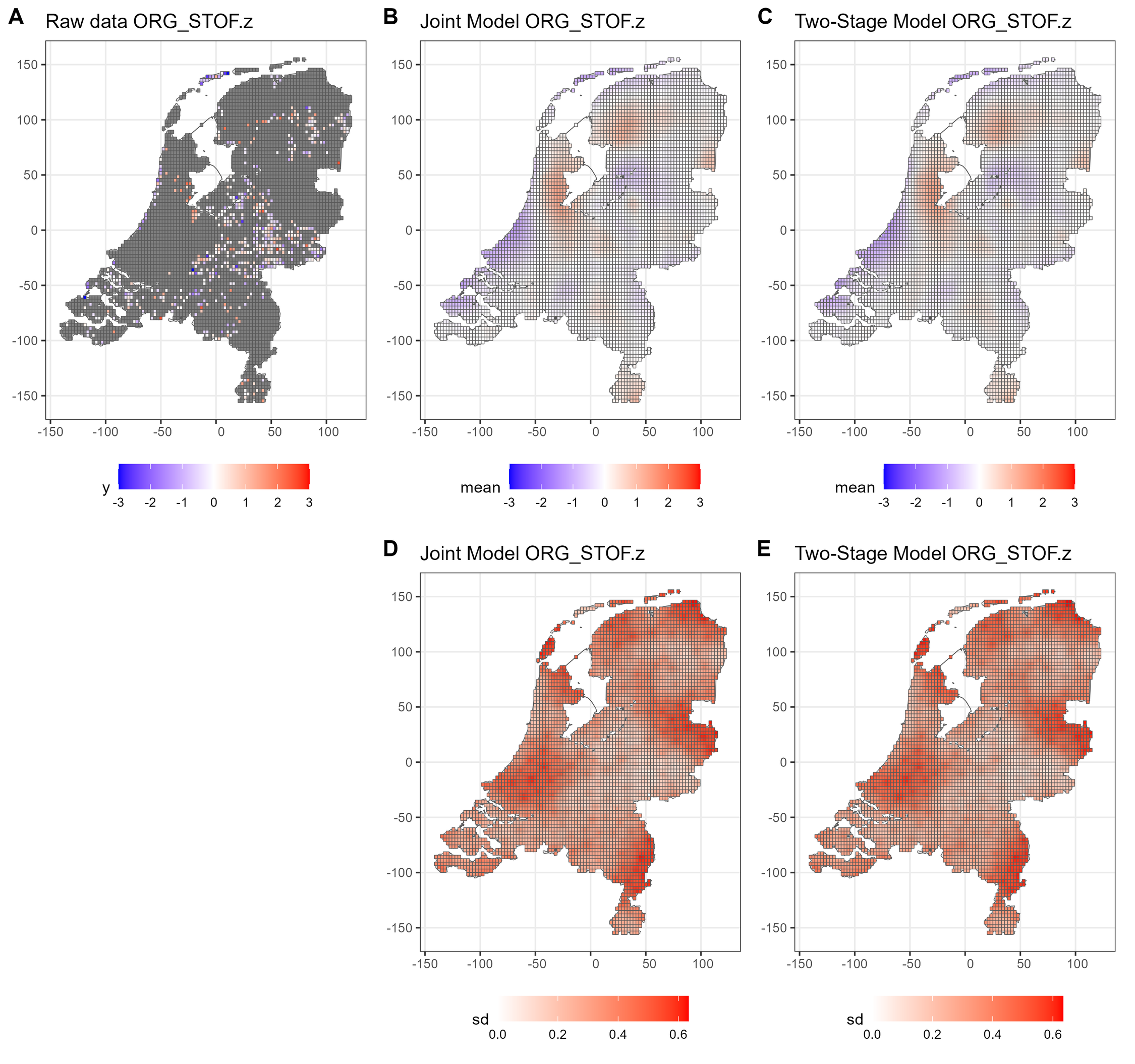

Supplement: S4 Fig — Left panel (A) is the observed organic matter mean value in each grid cell. The top row middle (B) and right (C) panels compare the mean predicted value in the joint and two-stage models. The bottom row middle (D) and right (E) panels compare the standard deviation, i.e. uncertainty, of the predicted value. Joint model uses Empetrum nigrum as the SDM species. (TIF) [file pone.0304942.s008.tif]

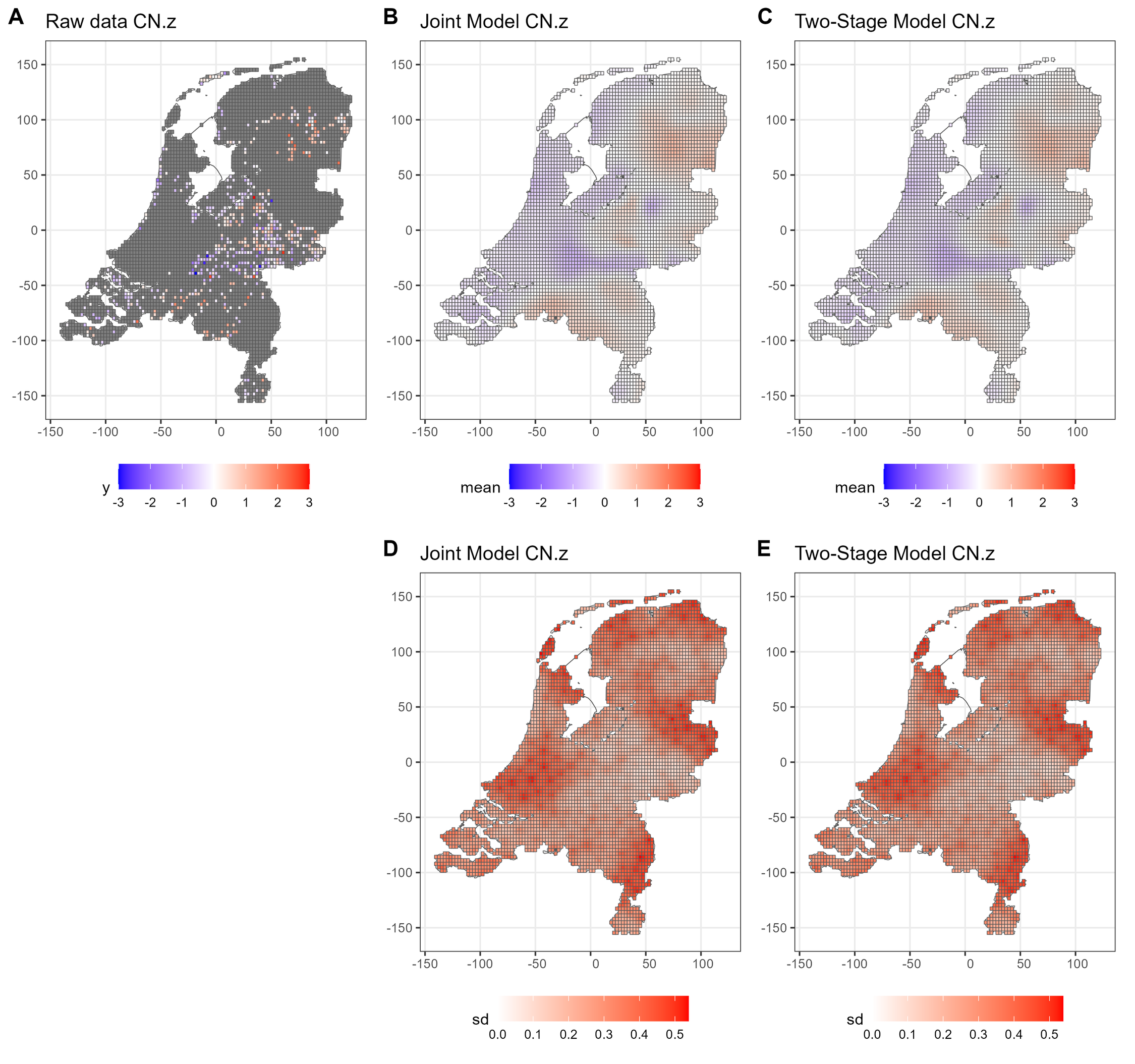

Supplement: S5 Fig — Left panel (A) is the observed C/N-ratio mean value in each grid cell. The top row middle (B) and right (C) panels compare the mean predicted value in the joint and two-stage models. The bottom row middle (D) and right (E) panels compare the standard deviation, i.e. uncertainty, of the predicted value. Joint model uses Empetrum nigrum as the SDM species. (TIF) [file pone.0304942.s009.tif]

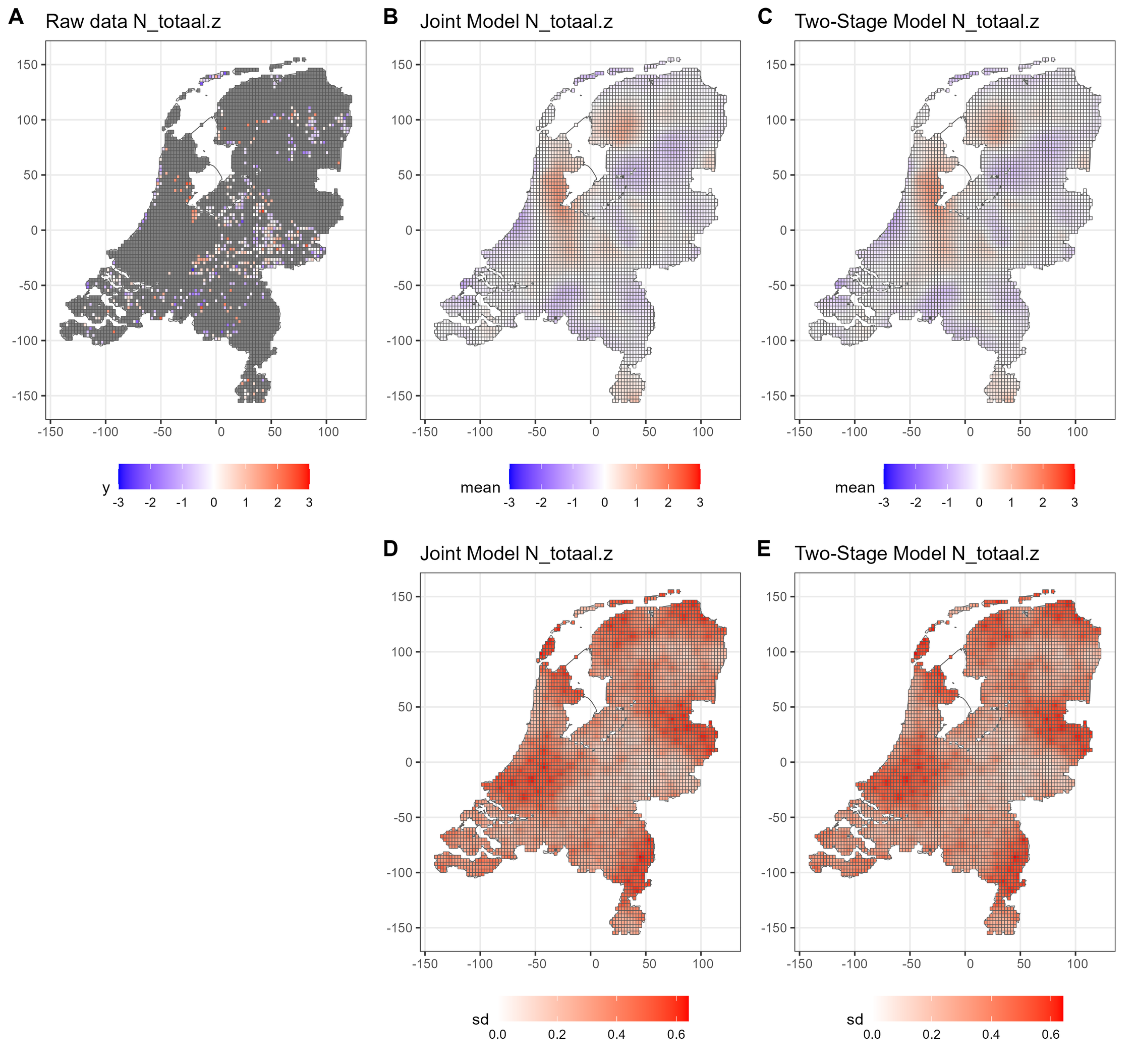

Supplement: S6 Fig — Left panel (A) is the observed total nitrogen mean value in each grid cell. The top row middle (B) and right (C) panels compare the mean predicted value in the joint and two-stage models. The bottom row middle (D) and right (E) panels compare the standard deviation, i.e. uncertainty, of the predicted value. Joint model uses Empetrum nigrum as the SDM species. (TIF) [file pone.0304942.s010.tif]

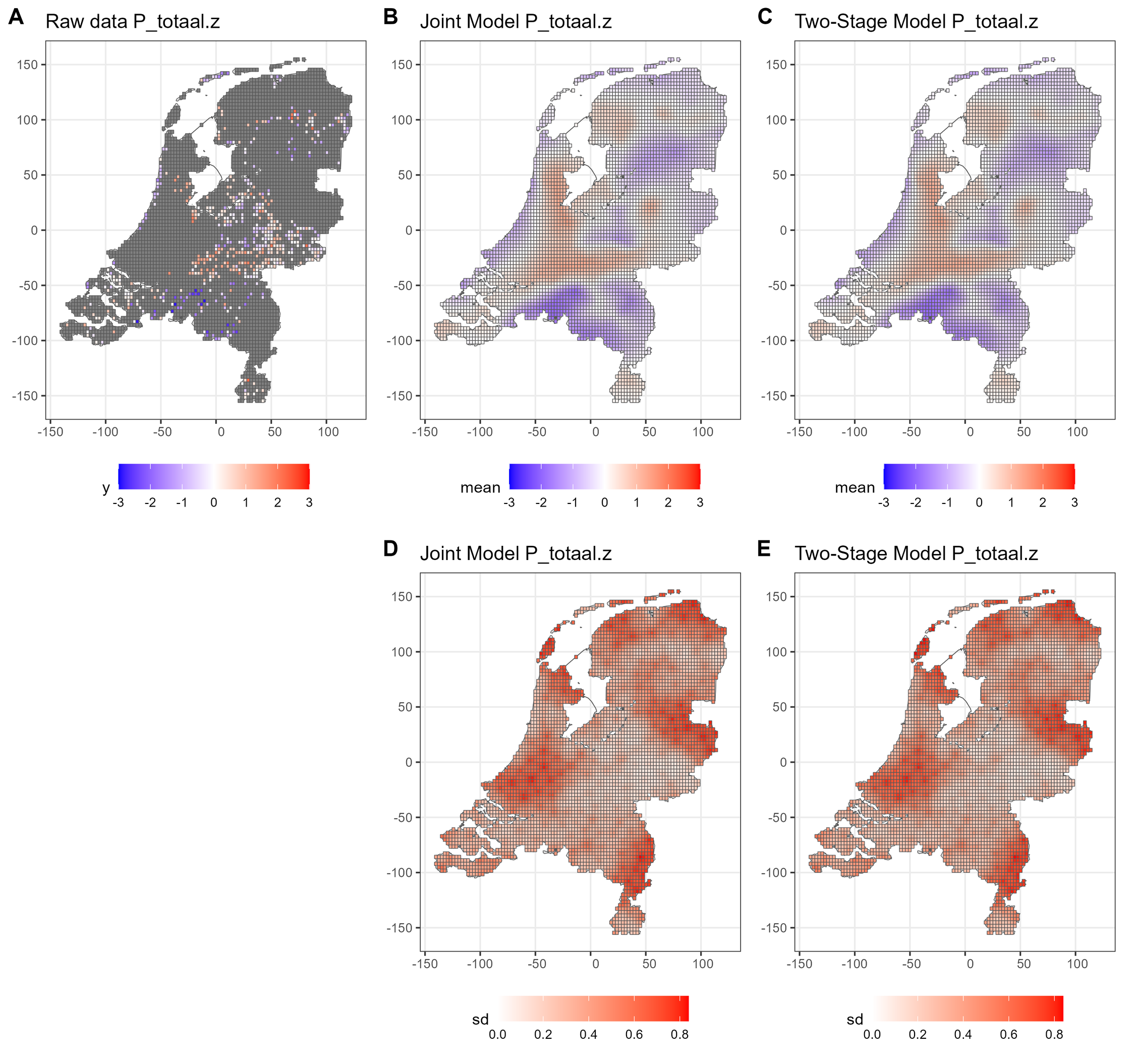

Supplement: S7 Fig — Left panel (A) is the observed total phosphorus mean value in each grid cell. The top row middle (B) and right (C) panels compare the mean predicted value in the joint and two-stage models. The bottom row middle (D) and right (E) panels compare the standard deviation, i.e. uncertainty, of the predicted value. Joint model uses Empetrum nigrum as the SDM species. (TIF) [file pone.0304942.s011.tif]

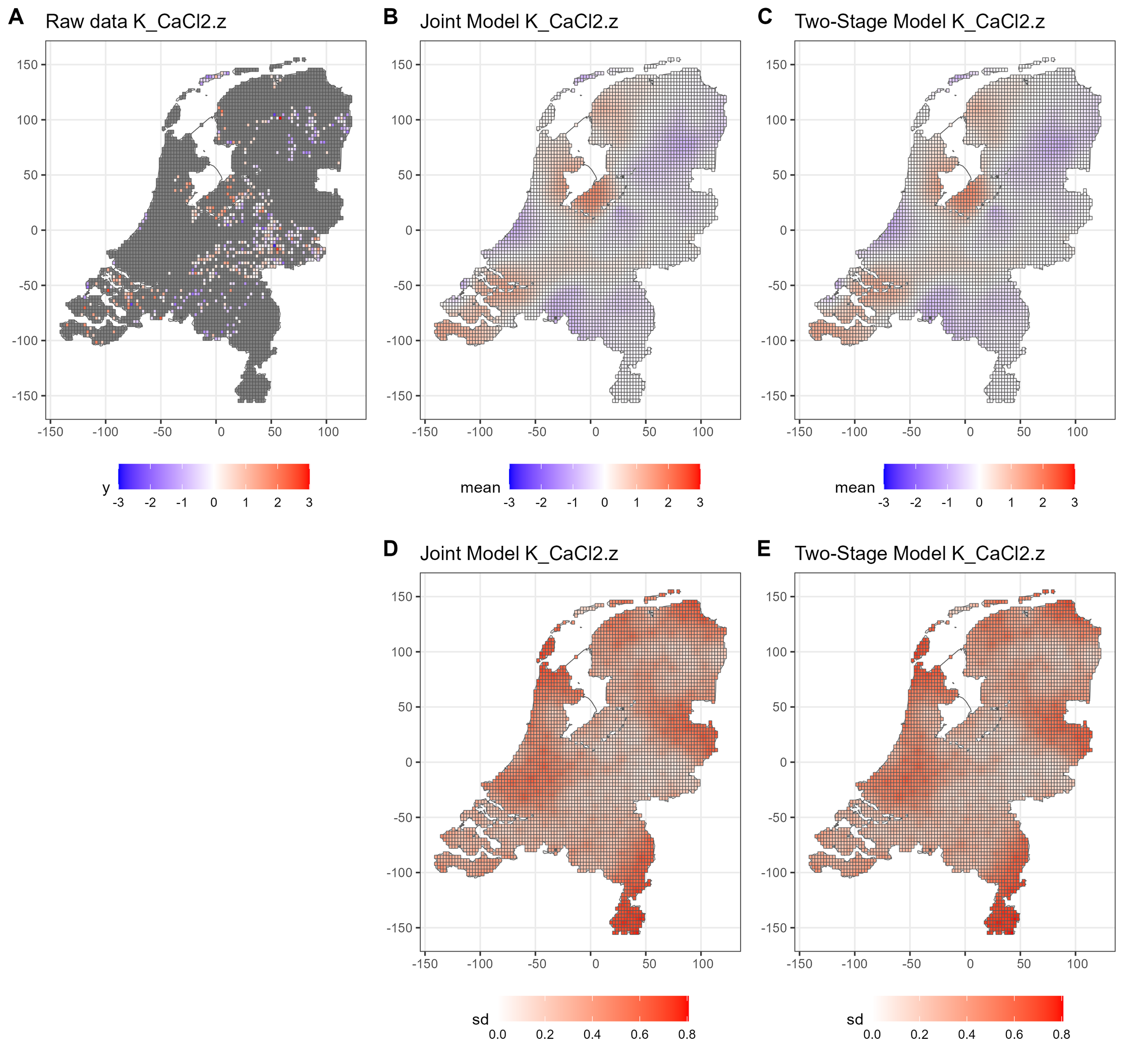

Supplement: S8 Fig — Left panel (A) is the observed total potassium mean value in each grid cell. The top row middle (B) and right (C) panels compare the mean predicted value in the joint and two-stage models. The bottom row middle (D) and right (E) panels compare the standard deviation, i.e. uncertainty, of the predicted value. Joint model uses Empetrum nigrum as the SDM species. (TIF) [file pone.0304942.s012.tif]

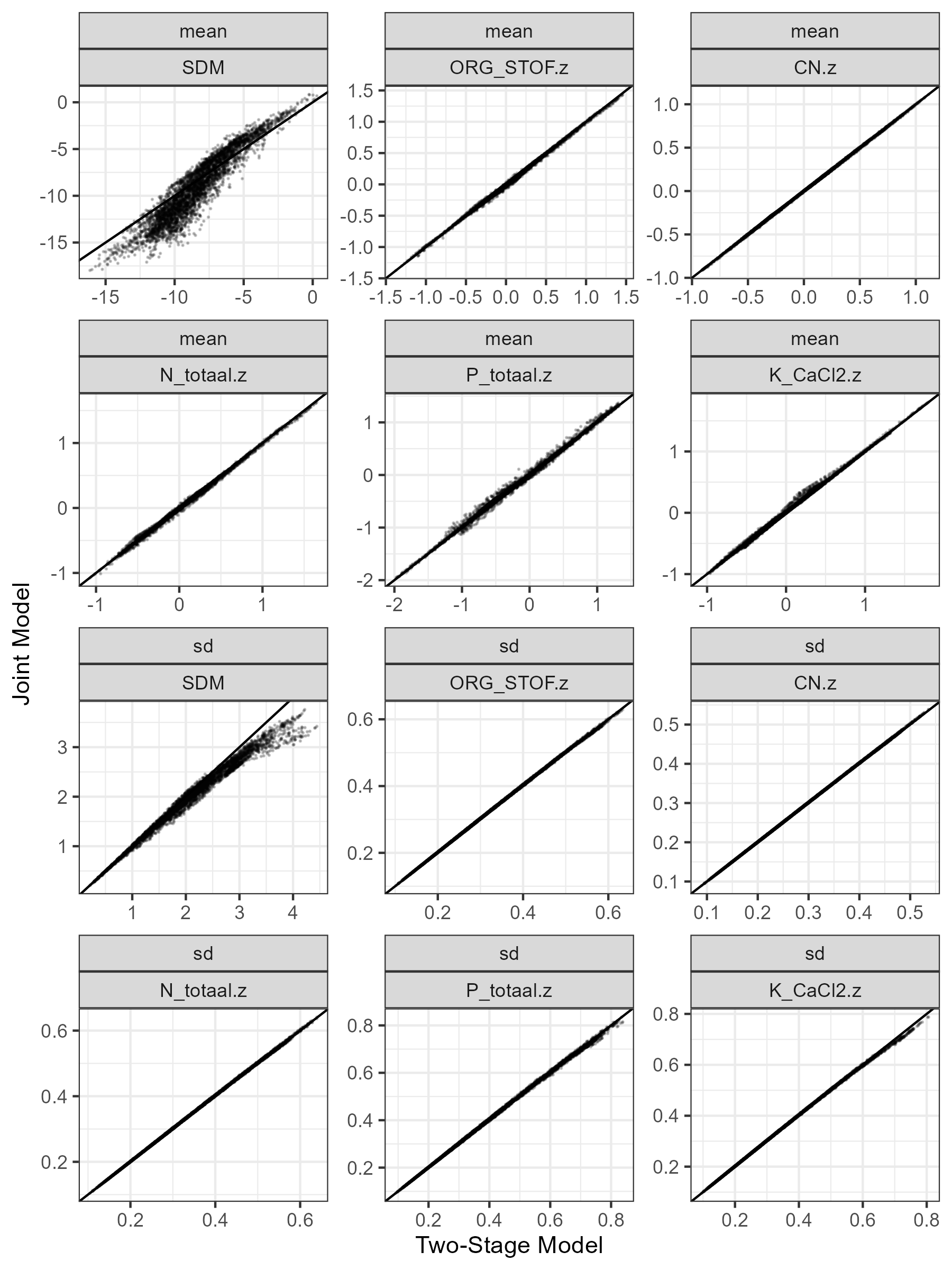

Supplement: S9 Fig — The top 6 panels illustrate the mean predicted value, while the bottom 6 panels present the standard deviation of the predicted value, i.e. uncertainty of the prediction. (TIF) [file pone.0304942.s013.tif]

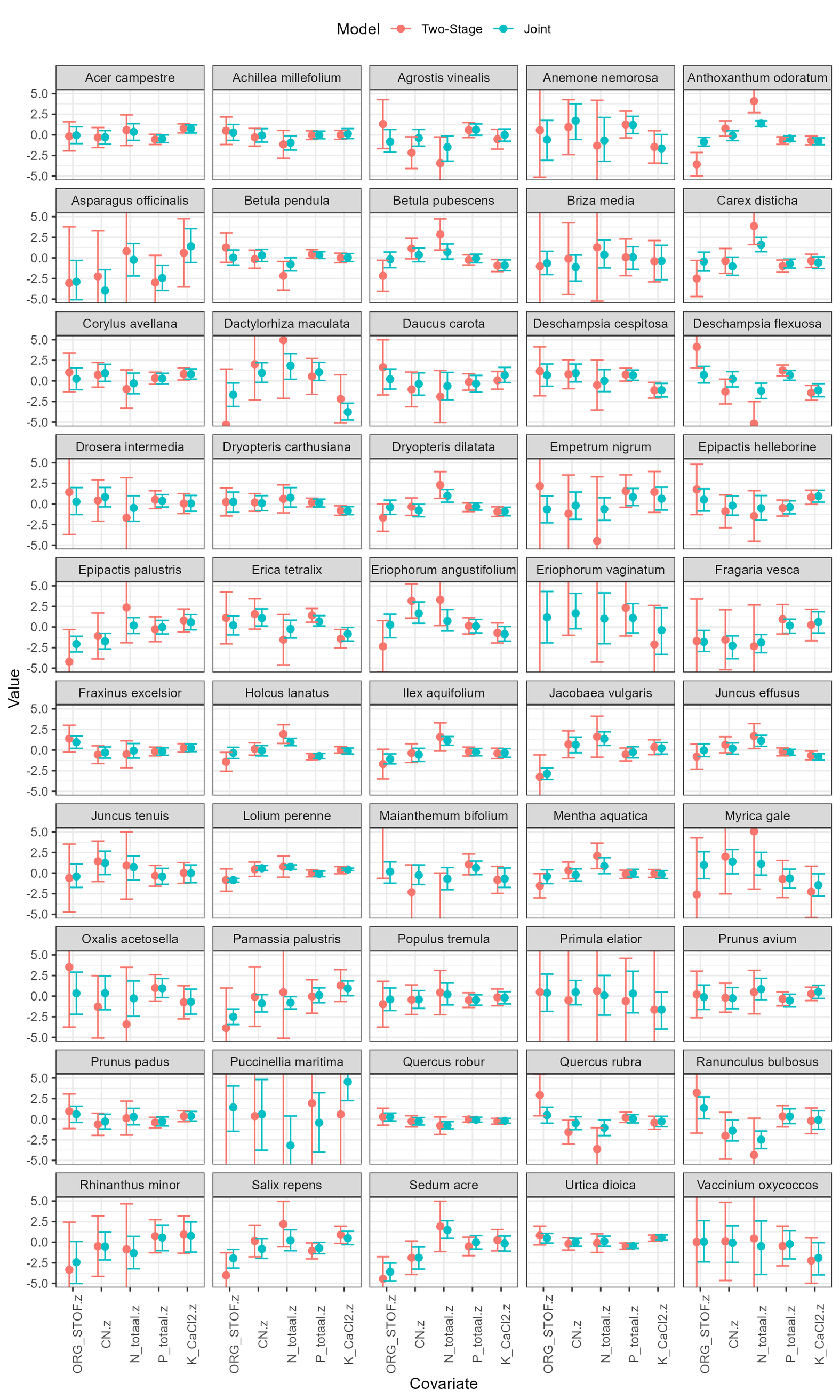

Supplement: S10 Fig — (TIF) [file pone.0304942.s014.tif]

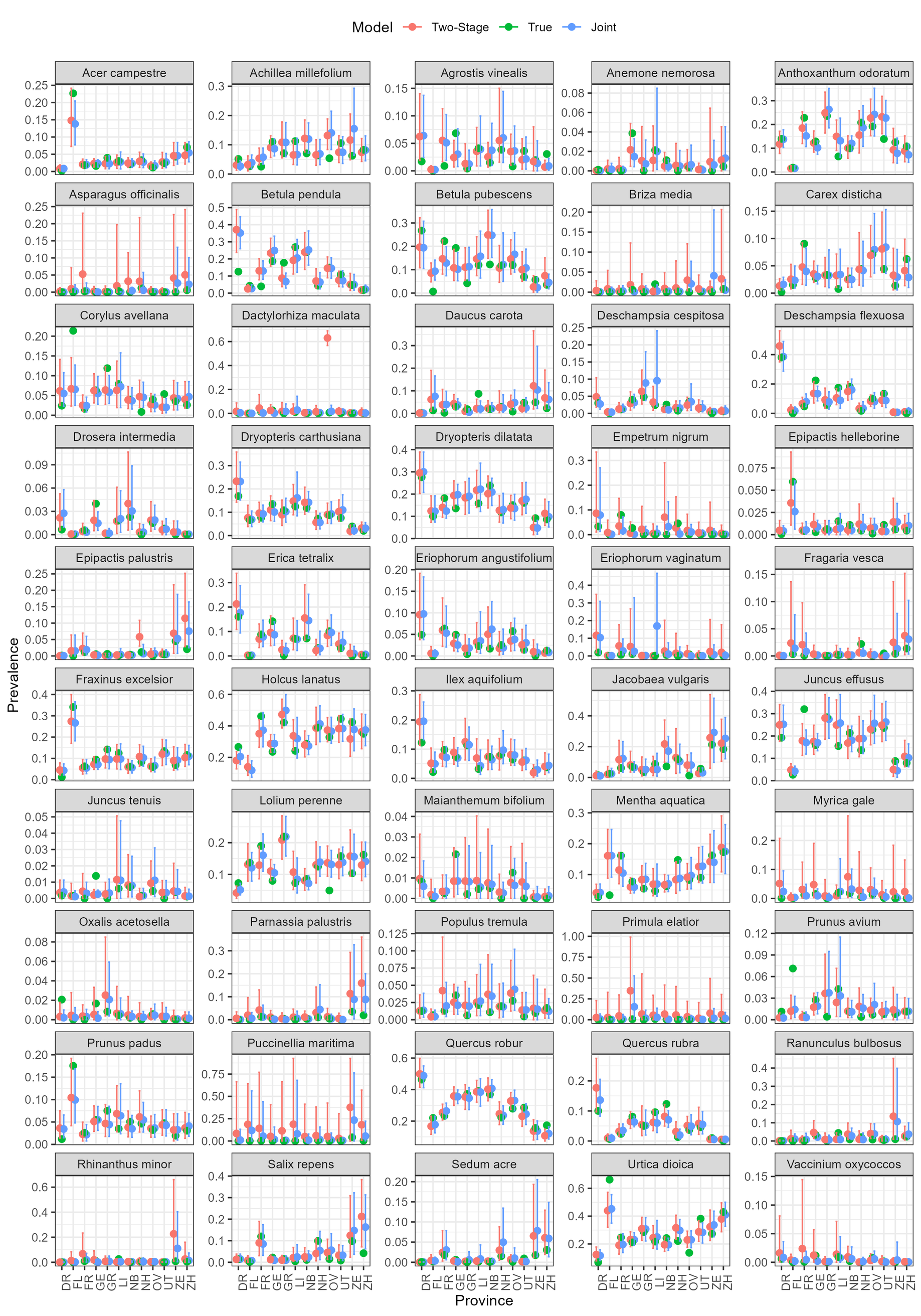

Supplement: S11 Fig — These predictions are compared with the true prevalence (green) in each province. (TIF) [file pone.0304942.s015.tif]

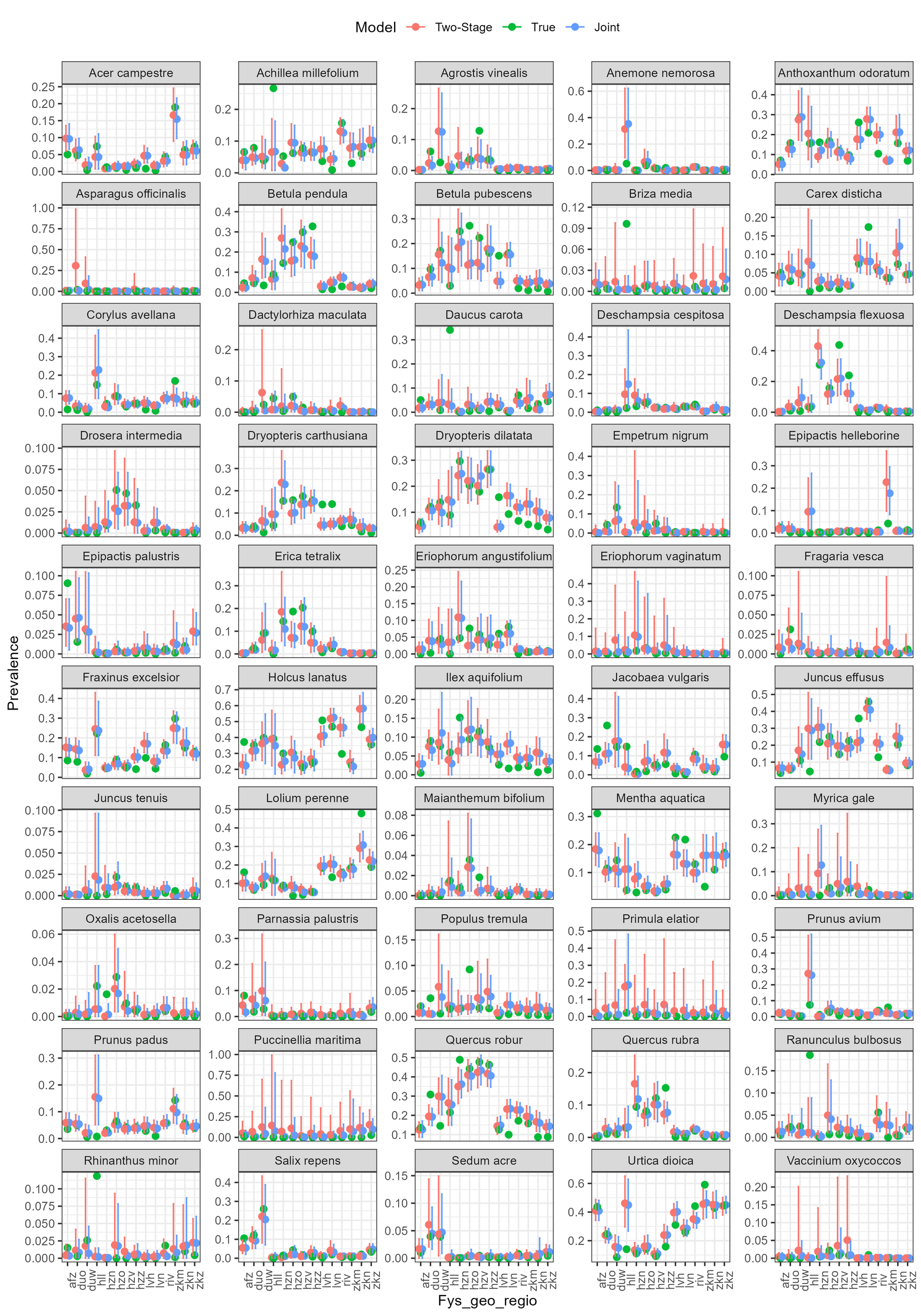

Supplement: S12 Fig — These predictions are compared with the true prevalence (green) in each FGR region. (TIF) [file pone.0304942.s016.tif]
